# A Review on Antibacterial Biomaterials in Biomedical Applications: From Materials Perspective to Bioinks Design

**DOI:** 10.3390/polym14112238

**Published:** 2022-05-31

**Authors:** Farnoosh Pahlevanzadeh, Mohsen Setayeshmehr, Hamid Reza Bakhsheshi-Rad, Rahmatollah Emadi, Mahshid Kharaziha, S. Ali Poursamar, Ahmad Fauzi Ismail, Safian Sharif, Xiongbiao Chen, Filippo Berto

**Affiliations:** 1 Department of Materials Engineering, Isfahan University of Technology, Isfahan 84156-83111, Iran; farnoosh.pahlevanzadeh@gmail.com (F.P.); remadi@cc.iut.ac.ir (R.E.); 2 Department of Biomaterials, Nanotechnology and Tissue Engineering, School of Advanced Technologies in Medicine, Isfahan University of Medical Sciences, Isfahan 81746-73461, Iran; setayeshmehr.m@gmail.com; 3 Advanced Materials Research Center, Department of Materials Engineering, Najafabad Branch, Islamic Azad University, Najafabad, Iran; 4 Biomaterials, Nanotechnology, and Tissue Engineering Group, Advanced Medical Technology Department, Isfahan University of Medical Sciences, Isfahan 81746-73461, Iran; ali.poursamar@amt.mui.ac.ir; 5 Advanced Membrane Technology Research Center (AMTEC), Universiti Teknologi Malaysia, Johor Bahru 81310, Johor, Malaysia; afauzi@utm.my; 6 Faculty of Engineering, Universiti Teknologi Malaysia, Johor Bahru 81310, Johor, Malaysia; safian@utm.my; 7 Department of Mechanical Engineering, College of Engineering, University of Saskatchewan, Saskatoon, SK S7N 5A9, Canada; xbc719@mail.usask.ca; 8 Department of Mechanical and Industrial Engineering, Norwegian University of Science and Technology, 7491 Trondheim, Norway

**Keywords:** antibacterial properties, chitosan, 3D printing, metallic ions

## Abstract

In tissue engineering, three-dimensional (3D) printing is an emerging approach to producing functioning tissue constructs to repair wounds and repair or replace sick tissue/organs. It allows for precise control of materials and other components in the tissue constructs in an automated way, potentially permitting great throughput production. An ink made using one or multiple biomaterials can be 3D printed into tissue constructs by the printing process; though promising in tissue engineering, the printed constructs have also been reported to have the ability to lead to the emergence of unforeseen illnesses and failure due to biomaterial-related infections. Numerous approaches and/or strategies have been developed to combat biomaterial-related infections, and among them, natural biomaterials, surface treatment of biomaterials, and incorporating inorganic agents have been widely employed for the construct fabrication by 3D printing. Despite various attempts to synthesize and/or optimize the inks for 3D printing, the incidence of infection in the implanted tissue constructs remains one of the most significant issues. For the first time, here we present an overview of inks with antibacterial properties for 3D printing, focusing on the principles and strategies to accomplish biomaterials with anti-infective properties, and the synthesis of metallic ion-containing ink, chitosan-containing inks, and other antibacterial inks. Related discussions regarding the mechanics of biofilm formation and antibacterial performance are also presented, along with future perspectives of the importance of developing printable inks.

## 1. Introduction

The human body provides an impressive potential for tissue regeneration; however, this potential is limited by factors including tissue type and the requirement for growth hormones for differentiation, and physical size (crucial defect) [1,2,3]. Any damage to a tissue bigger than this crucial size requires the utilization of alternative aid, which has led to the development of tissue engineering (TE) and/or regenerative medicine (RM). For this, scaffolds are typically used to act as a support for cells’ functions and growth, so as to generate new tissue, thus playing an important role in TE and RM [4,5,6,7,8]. Numerous studies have been conducted to create scaffolds by employing such traditional approaches as porogen leaching and gas forming [8,9,10,11,12], but these approaches suffered from many limitations, including low mechanical strength, residual solvents in scaffolds, no or minimal control over pore size and interconnectivity, large energy consumption, prolonged timescales, and the use of cytotoxic solvents [13,14]. To overcome these limitations, an emerging approach known as three-dimensional (3D) printing has been developed based on the principle of additive manufacturing, where the solution of one or more biomaterials (referred to as ink) is deposited or printed, layer by layer, to create scaffolds with a 3D structure [13,14].

Stereolithography (SLA), as the first 3D printing method, was invented by Charles Hull in 1984. In 1988, bioprinting was first developed, while Klebe used Hewlett-Packard (HP) to deposit cells by cytoscribing technology using fibronectin solution [15]. This modern method was evolving rapidly until, in 2002, the first extrusion-based bioprinting was introduced by Landers et al. and commercialized as “3D-Bioplotter”. Wilson and Boland modified the HP printer and developed the first inkjet bioprinter in 2003 [16]. These 3D printing techniques allow for the creation of customized patient-specific designs with a large scale of reproduction, as well as the incorporation of cells, if needed, to form cell-laden matrices or constructs of various TE applications [17,18,19,20,21,22,23,24].

The synthesis of inks is critical to the success of 3D printing scaffolds for TE. An ink should have appropriate properties, including printability, mechanical properties (or stability), and biological properties (including degradation/insolubility in physiological solutions, cytocompatibility, and non-immunogenicity [1,2,3,25]). Despite numerous studies that have been reported on ink synthesis and optimization [26,27,28,29,30,31] for printing, the antibacterial behavior of inks has barely been investigated and documented. Bacterial infections occurred in biomedical implants and TE scaffolds, and devices have become a severe issue in recent years, resulting in implantation failure and/or escalating probability of revision surgery. The creation of biofilm on the material surfaces triggers these bacterial infections [32]. Since the first synthesis of penicillin in 1928, various new antibiotics have been developed for remedying microbial infections, owing to its great bactericidal action and minimal toxicity to mammalian cells. Considering that the growth rate of new antimicrobial drugs can barely keep up with the advancement of bacterial resistance, it is becoming progressively clear that the "post-antibiotic era" is on the horizon. Annually, more than 700,000 patients pass away due to drug-resistant germs; as a result, there is a vital requirement to generate new antibacterial biomaterials to enhance bacterial selectivity and lessen antibiotic resistance [33]. In this regard, natural antibacterial materials such as Ag, Au, and some oxides including ZnO, TiO_2_, and MgO have been utilized as additives to form composites with antibacterial characteristics [34]. To effectively combat these infections, a number of approaches, including surface chemical modifications and the compositional differentiation of implants and TE scaffolds, have been developed and reported [35]. With the latest advance in 3D printing, the hunt to design and generate antibacterial ink for printing stands out and remains to be addressed by research [36,37,38,39,40]. Over the past years, considerable progress has been made in the development of printable inks for 3D printing and tissue engineering. Here, we review this progress with an emphasis on the essentiality of antibacterial features in biomaterials, principles, and strategies to accomplish biomaterials with anti-infective properties, and the synthesis of metallic ion-containing inks, chitosan-containing inks, and other antibacterial inks for 3D printing, as well as some future perspectives of importance in this field.

## 2. Why Antibacterial Features Are Necessary for Biomaterials?

**Figure 1:**
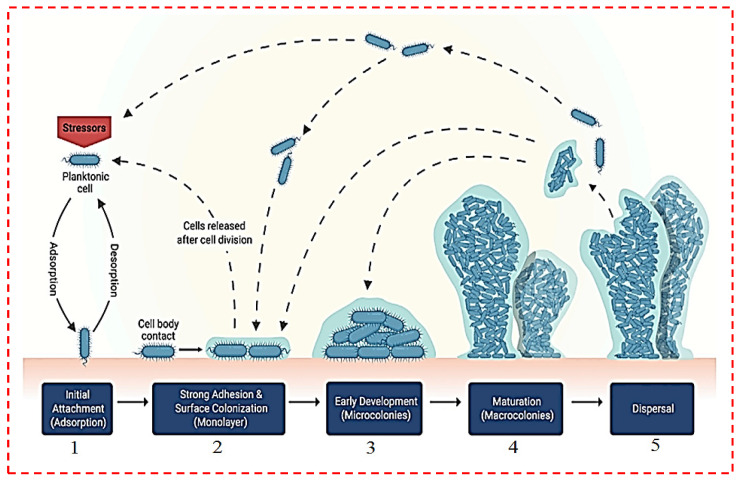
Schematic of the formation of a single bacterial biofilm on a solid surface [25].

Biomaterials play an essential function in disease treatment and healthcare improvement. The diversity, function, and wide variety of biomaterials employed worldwide have improved considerably in recent years. Nevertheless, the attachment of harmful microorganisms to biomaterial surfaces, leading to biofilm growth, continues to be a major issue that seriously restricts the functional use of these systems [41]. Figure 1 depicts the development of a biofilm, including initiation attachment of bacteria, colonization, early development, maturation, and disintegration [25]. Under typical flow conditions, the biofilm functions as a physical defense obstacle towards phagocytic predation and inhibits cell detachment, along with a selective permeability barrier [25,42]. Physical and chemical permeability allow the productive transfer of organic molecules and ions to cells positioned at a distance from the biofilm’s surface, binding and concentrating nutrients alongside cells to ensure the cell population’s survival [17]. The identical procedure inhibits the propagation of bacteria-damaging chemicals, including systemic antibiotics employed to deal with postsurgical infections and antibacterial drugs seeping out of the biomaterial matrix. In other cases, the inability of conventional antibiotics to generate direct contact with the bacteria leads to merely partial reductions of the infection, leading to chronic infections and deadly outcomes. Subsequently, it is not surprising that dealing with biomaterial-associated infections might be challenging, regularly necessitating the physical (surgical) removal of the infected device [41]. Due to the improvements made in aseptic treatments, environmental sterility control, and peri-surgical antibiotic prophylaxis in recent years, anti-infective biomaterials have progressively become a primary approach for avoiding medical tool-connected infections [43,44]. Antibacterial biomaterials are employed for the manufacture of medical tools, with anti-infective supplementary bioactive characteristics that find out their resistance to infections. They can likewise be utilized to render medical compounds, whose main function is to prevent, treat, or reduce infections, to extend the biomedical fields [45].

## 3. Strategies for Achieving Antibacterial Biomaterials

To accomplish biomaterials with anti-infective properties, a number of principles and strategies have been uncovered and/or developed [44]. This section presents a brief overview of the most common approaches based on two principles—aiming to lower the susceptibility of medical tools to bacterial colonization and infection or using materials with antibacterial properties.

### 3.1. Surface Treatment with Bacteria Repelling and Antiadhesive Substances

Bacterial attachment to the surface is the initial phase in the development of foreign-body infections. If the surface is treated with bacterial repelling substances, bacteria are incapable of sticking and hence, colonization is certainly unattainable. Bacterial attachment on biomaterial surfaces is considered to take place by a wide range of mechanisms; a number of them are transversal to all microbial species, while others are specific to a species or a particular type of strain. As a result of physicochemical surface connections, the previous procedures lead to passive adsorption of bacterial cells on a solid surface. The latter are active attachment mechanisms mediated through bacterial structures and referred to as bacterial attachments [46,47]. Hydrophilic, extremely hydrated, non-charged surfaces, including those generated through specific polymer brush coatings, are effective surfaces with low attachment capability. These surfaces seem to restrict bacterial interaction and feasible attachment on the material surface. When developing new coatings, the approach through which the coating is positioned and stabilized on the biomaterial to be resurfaced is vital. Recent approaches to functional antifouling coatings depend on a number of strategies, such as self-assembled mono- or multilayer manufacturing, polymer brushes, surface grafting, zwitterionic polymers, and hydrogels. In addition to chemistry, the topography of the surface could be altered to restrict bacteria attachment. Rough and porous interfaces are actually exhibited to boost bacterial interactions and escalate microbial attachment. Reducing the size of the nanometric scale has been revealed in vitro to be related to the minimum attachment of both Gram-positive and Gram-negative bacteria [48].

### 3.2. Materials with Antibacterial Properties

There are various materials that have intrinsically antibacterial properties for biomedical applications. These materials include metals (e.g., Ag, Zn, and Cu), polymeric materials (e.g., CS) and their potentiated derivatives, as well as ceramics (e.g., ZnO, MgO, and TiO_2_) [18,25,44]. Due to their bactericidal abilities, these materials have been widely utilized in various biomedical applications, as summarized in Table 1.

#### 3.2.1. Antibacterial Activity of Copper

**Figure 2:**
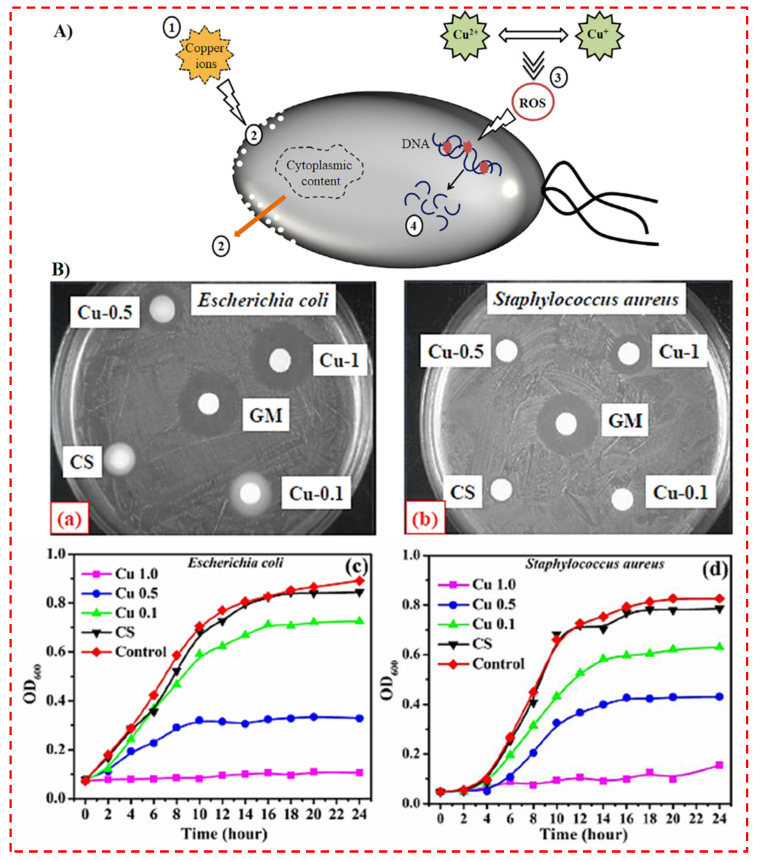
(**A**) The schematic illustration of the killing action involved in the antibacterial activity of copper. Copper ions are released from the doped biomaterial (1) and cause membrane damage leading to a loss of cytoplasmic content (2). Then, the production of reactive oxygen species (ROS) (3) causes DNA fragmentation (4) and cell death [26]. (**B**) Inhibition zone against (**a**) *E. coli* and (**b**) *S. aureus*, growth inhibition curve in TGY broth against (**c**) *E. coli* and (**d**) *S. aureus* [50].

Even though the mechanism’s base metallic nanostructures’ biocidal activity is not entirely comprehended, three hypotheses have been acknowledged and described in the literature: (1) the accumulation of NPs in the bacterial membrane layer altering its permeability, with the generation of membrane biomolecules, and (2) the generation of reactive oxygen species (ROSs) with subsequent oxidative damming, as shown in Figure 2A [26]. A couple of studies have been published, especially on the mechanism of the bactericidal activity of CuNPs, which proposed that Cu^2+^ ions coming from the NPs may interact with phosphorus and sulfur-containing biomolecules, including DNA and protein, damaging their structures, and hence disrupting biochemical functions [117,118,119]. Copper’s antibacterial characteristics are the main basis for its utilization for biomedical purposes. In this regard, Sahithi et al. [49] produced nHAp and nHAp–Cu for BTE applications. They found that whereas nHAp did not possess antibacterial action when it was soaked in copper, it did have antimicrobial activity in both Gram-positive and negative pathogens. Mishra et al. [50] also developed a multipotent wound dressing material out of copper ion- (Cu^2+^) infused microporous CS-polyethylene glycol sheets. In comparison to CS films, their antibacterial tests pointed out superiority in suppressing biofilm growth. The antibacterial action of the films was examined on Gram-negative (*E. coli*) and Gram-positive (*S. aureus*) bacteria, employing the modified disc diffusion assay as shown in Figure 2B [50]. The CS films indicated no IZ in the disc diffusion assay, indicating remarkably low antibacterial performance after neutralization, which is caused by the non-protonated form of CS. All Cu^2+^-incorporated films presented outstanding antibacterial performance towards both tested bacteria, with increasing IZ as a function of Cu^2+^ content. Based on the bulk of the study results (Table 1), copper could be considerably more productive at generating antibacterial effects on Gram-positive compared to Gram-negative bacteria.

#### 3.2.2. Antibacterial Activity of Silver

**Figure 3:**
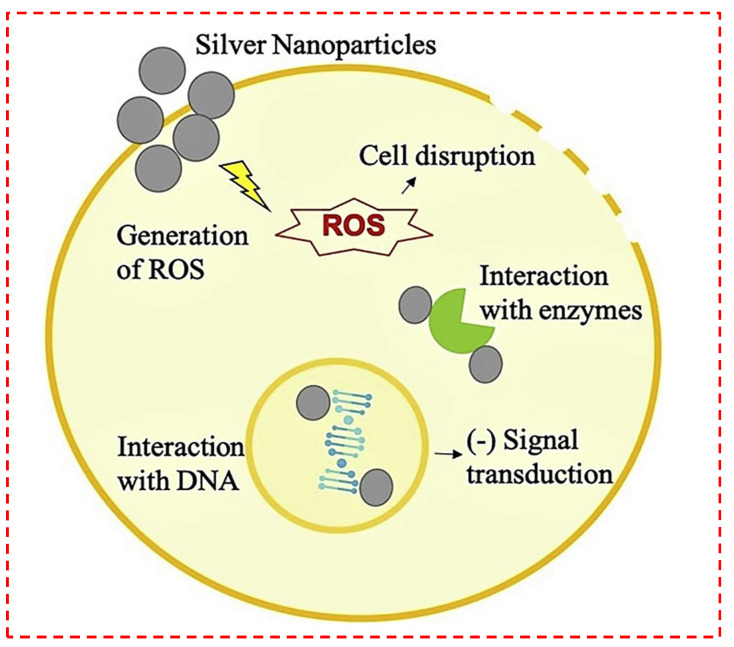
The schematic illustration of feasible antibacterial mechanisms of Ag nanoparticles (AgNPs) [46].

Despite the fact that silver and associated compounds were employed to cure ailments and disease, their performance as antibacterial agents was not identified until the late 1800s [120]. Silver is actually an antibiotic with a wide range. In this regard, Suresh et al. [19] reported biofabrication of silver nanocrystallites using Shewanella oneidensis, which indicated favorable antibacterial properties for Gram-negative and Gram-positive bacteria [19]. Resistance to silver-based compounds is rare in organisms, especially Gram-negative bacteria [20]. Bacteria destroyed by silver could possess 10^5^–10^7^ Ag^+^ molecules per cell, which is the same order of magnitude as the predicted number of enzyme-protein molecules per cell. Metallic silver is fairly inert chemically; however, its interaction with water on the skin’s surface and wound liquids generates silver ions and its biocidal components. The possible routes of antibacterial activity of AgNPs are portrayed diagrammatically in Figure 3 [46]. The positive charge of Ag^+^ interacts with the negative charge on the cell wall membrane of bacteria, leading to modifications in cell wall membrane shape and an enhancement in cell permeability or leakage, leading to cell fatality [121]. AgNPs possess a greater affinity for interacting with phosphorous and sulfur-containing biomolecules identified in external (membrane protein) and intracellular (DNA bases and protein) components; these biomolecules impact cell division, taking in oxygen, and, eventually, cell survival [120]. Other reports have found that Ag^+^, which has a nitrogen and sulfur affinity, can prevent and destabilize protein structures by connecting to thiol and amino groups [122]. The connection of nanoparticles with thiol groups could be responsible for the generation of ROS, which suppress respiratory enzymes and, for that reason, cause fatality [120]. Silver ions are antibacterial due to the fact they interact with the peptidoglycan cell wall membrane and the plasma membrane, and they likewise obstruct bacterial DNA reproduction by interfering with sulfhydryl groups within proteins [121]. Silver has been broadly utilized in TE and wound treatments as a result of its antimicrobial features [61,62,63,79].

In this respect, Augustine et al. [61] depicted electrospun PCL membranes infused with biosynthesized AgNPs as antibacterial wound dressings. Their findings depicted that the fabricated membrane-encapsulated AgNPs presented great antibacterial action towards both *S. aureus* and *E. coli*. In accordance with the results, neat PCL membranes had no effectiveness towards the bacteria examined. The inhibitory zone diameter of the PCL membrane-encapsulated 1 wt. % AgNPs was 11.6 ± 0.5 and 7.9 ± 0.6 mm towards *S. aureus* with *E. coli*, respectively. Similarly, in another work, Saini et al. [62] fabricated Ag-HA-based antibacterial 3D gelatin/alginate/PVA scaffolds for BTE purposes. Antimicrobial activity examination for fabricated Ag-HA and Ag-HA incorporated with a gelatin-alginate-PVA cryogel scaffold were conducted towards *Bacillus* and *E. coli*, implying that both scaffolds presented bacteria destruction characteristics [62].

#### 3.2.3. Antibacterial Activity of Gold

**Figure 4:**
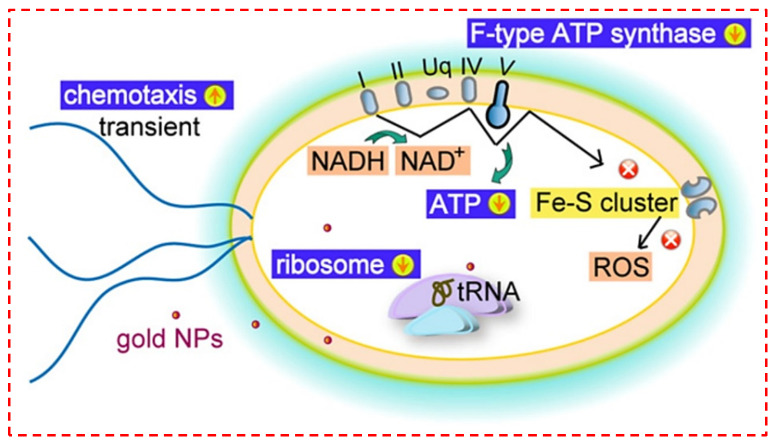
The schematic presentation of feasible antibacterial mechanisms of Au nanoparticles (AuNPs) [47].

The utilization of gold nanoparticles (AuNPs) in the treatment of bacterial infections has appeared as a feasible choice; nevertheless, the mechanism accountable for bacterial cell lysis continues to be unidentified [123]. When AuNPs get into cells, they might occasionally break down cell walls. Consequently, they can assist some antibiotics incapable of passing through the cell wall membrane barrier. Findings have pointed out that inhibition by AuNPs is induced via direct contact, which leads to cell wall infiltration, instead of by the creation of ROS. Coradeghini’s group speculated that the absorption of AuNPs into cells amplified with particle size. They found that particle size has an influence on antibacterial functions. Smaller NPs have the capability to infiltrate cells. By acting on the surface, larger NPs trigger cell lysis and death [124]. Nevertheless, no comprehensive, detailed description of these systems exists as of yet. Extremely dispersed NPs have a higher contact area, and their antibacterial action is distinct. Nanoparticles’ main ways of action consist of injuring the cytoderm, destroying cytomembranes, and transforming the inside of bacterial cells. Some NPs modified with a photocatalytic metal, on the other hand, depend on light stimulation to generate free radicals for antibacterial reasons. Figure 4 depicts the essential antibacterial performance of AuNPs [47]. It was suggested that two mechanisms are accountable for the antibacterial performance of AuNPs. First, via breaking down the membrane layer, which prevents ATPase action, and next, via suppressing the subunits of ribosomes for tRNA binding. As opposed to the previously mentioned antibacterial nanoparticles, AuNPs demonstrated ROS-independent antibacterial performance.

AuNPs are employed in biomedical use, including TE and wound healing. For example, Prakash et al. [82] fabricated CS/PVA/GO/HA/Au films using a gel casting approach for possible orthopedic application. The zone of inhibition for CS/PVA/HA/Au composite film attained from all the assessments was higher compared to the CS/PVA/HA film. Furthermore, CS/PVA/GO/HA/Au film exhibits the best antibacterial performance [82]. AuNPs of various shapes (rods and spheres) and surfaces were encapsulated in hydrogels in Mahmoud et al.’s [89] study. Hydrogels of polyethylene glycol (PEG)-gold nanorods (AuNRs) and cationic poly allylamine hydrochloride (PAH)-AuNRs exhibited outstanding wound healing properties upon topical application on wounds employing an animal model. Moreover, hydrogels of PEG-AuNRs and PAH-AuNRs displayed an effective role in in vitro antibacterial performance towards *S. aureus* and *P. aeruginosa* and demonstrated marvelous wound healing characteristics regarding topical applications on wounds utilizing an animal model. In addition, hydrogels of PEG-AuNRs and PAH-AuNRs displayed potent in vitro antibacterial performance towards *S. aureus* and *P. aeruginosa* [89]. Based on the studies (Table 1), it seems that Au could be one of the suitable antibacterial metallic ions regarding the contribution to TE and wound dressing management.

#### 3.2.4. Antibacterial Activity of Zinc Oxide

**Figure 5:**
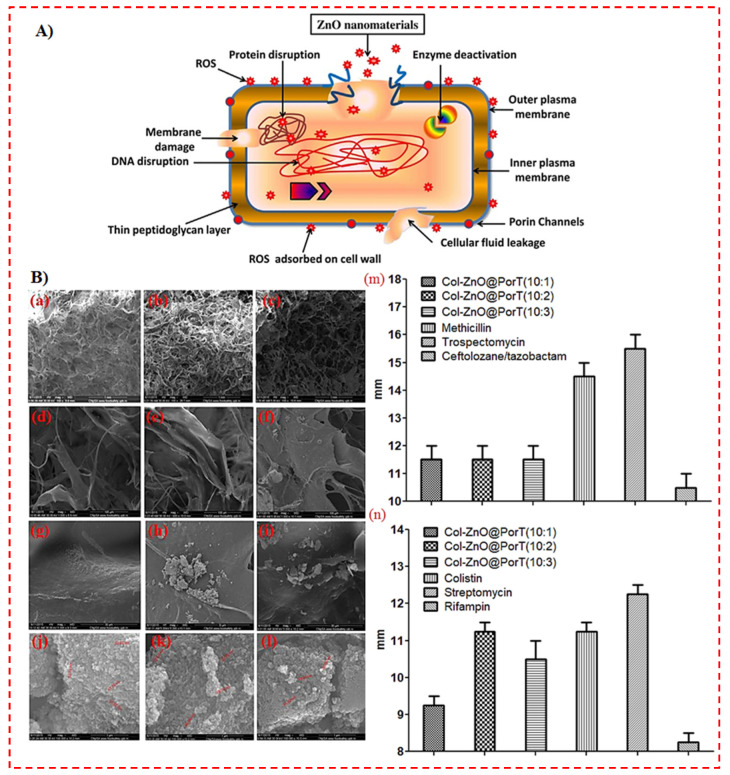
(**A**) The schematic illustration of Gram-negative bacteria cell damage through the production of reactive oxygen species (ROS) [40], and (**B**) scanning electron microscopy (SEM) micrograph obtained at various magnifications, ×100 (**a**–**c**), ×1000 (**d**–**f**), ×5000 (**g**–**i**), and ×100.000 (**j**–**l**), of the wound dressing, fabricated at the three diverse collagen (Col)/orange oil-functionalized zinc oxide NP (ZnO@PorT) mass ratios: 10:1 (**a**,**d**,**g**,**j**), 10:2 (**b**,**e**,**h**,**k**), and 10:3 (**c**,**f**,**I**,**l**). The diameter of the inhabitation zone (IZ) growth (measured at 20 h soaking duration at 37 °C) was created by different Col-ZnO@PorT dressings, and through typically utilized antibiotics towards *S. aureus* (**m**) and *E. coli* (**n**) strains [91].

The antibacterial performance of zinc oxide nanoparticles (ZnONPs) has attained considerable interest worldwide, specifically by rendering nanotechnology to fabricate particles in the nanometer area [125]. Despite the fact that the antibacterial performance of ZnONPs is referred to several issues, the exact toxicity mechanism is not entirely illuminated and still questionable, as there are some concerns within the range of antibacterial performance demanding profound descriptions. Unique mechanisms suggested in the literature are outlined as follows: direct contact of ZnONPs with cell walls, leading to the destruction of bacterial cell integrity, the release of antimicrobial ions, primarily Zn^2+^ ions, and ROS creation, as shown in Figure 5A [40]. Nevertheless, the toxicity mechanism varies in different solutions, as the species of dissolved Zn could alter based on the medium features besides the physicochemical characteristics of ZnONPs [125,126]. It is worth noting that ZnONPs have been employed in biomedical fields. For instance, Varaprasad et al. [90] fabricated ZnONPs by the precipitation approach and subsequently impregnated them appropriately over cellulose fibers via a sodium alginate (SA) matrix. The antimicrobial effectiveness of the ZnONPs–SA cellulose fibers (ZnO-SACNF) was tested toward *E. coli*. The IZ for all the fibers was identified to be in the range of 2.1–3.6 mm. Based on the standard antibacterial test “SNV 195920-1992”, specimens exhibiting more than 1 mm microbial zone inhibition are usually regarded as good antibacterial agents [90]. In another study, Balaure et al. [91] synthesized bioactive dressings consisting of collagen and zinc oxide 3D scaffolds. Morphological details of the Coll/ZnO nanocomposites were demonstrated through SEM images, as shown in Figure 5B (a–l), and the diameters of the growth IZ for the composites are presented in Figure 5B (m,n) [91]. As it can be seen in Figure 5B, at the lowest amount of ZnO@PorT, almost all of the NPs were encapsulated in the collagen matrix. The greater tendency of NPs to form clusters is obviously visible. The increased IZ of the nanostructured wound dressings is suitable and similar to the diameter of the IZ attained for tested antibiotics (Figure 5B (m,n)) [91].

#### 3.2.5. Antibacterial Activity of Titanium Dioxide

**Figure 6:**
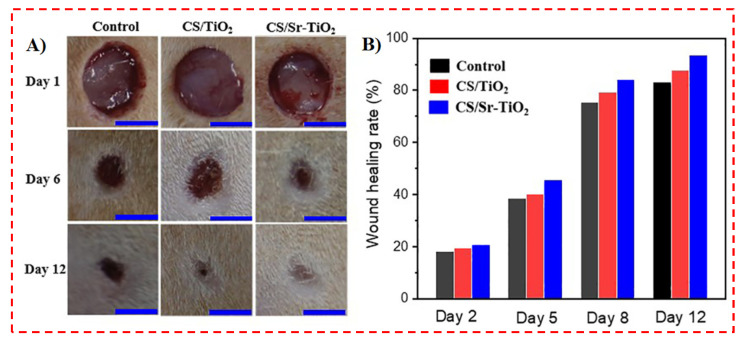
(**A**) In vivo evaluation of control, chitosan (CS)/titanium dioxide (TiO_2_), and CS/Sr-TiO_2_ nanocomposite as wound dressing, scale bar: 5 mm, and corresponding wound healing rate (**B**) [102].

Titanium dioxide nanoparticles (TiO_2_NPs) are essentially the most examined materials in the area of antimicrobial use relating to their distinct capabilities, including bactericidal photocatalytic activity, safety, and self-cleaning properties. The mechanism referred to the antimicrobial action of TiO_2_ is typically connected to ROS, with great oxidative potentials created within band gap irradiation photo-induced charge in the existence of O_2_. ROS have an impact on bacterial cells through various mechanisms, which result in cell death [127]. Chen et al. [102] used TiO_2_ to heal joint wounds, employing polymeric dressings of CS/Sr-doped TiO_2_ with great antibacterial activity. They tested the wound healing properties of CS/Sr-TiO_2_ in rabbit joint wounds. Their results confirmed that CS/Sr-TiO_2_ wound dressing was capable of attaining a great wound healing rate of around 93% after 12 days, as shown in Figure 6 [102]. In addition, Son et al. [103] also fabricated CS/PVA nanofibers with antibacterial behavior via the electrospinning of a CS/PVA solution with a small content of AgNO_3_ and TiO_2_. Their nanofibers presented antibacterial performance of 99 and 98% towards *S. aureus* and *E. coli*, respectively [103]. Generally, TiO_2_ is presented as a favorable material for biomedical use, particularly for wound healing and TE [103,104,105].

#### 3.2.6. Antibacterial Activity of Chitosan

**Figure 7:**
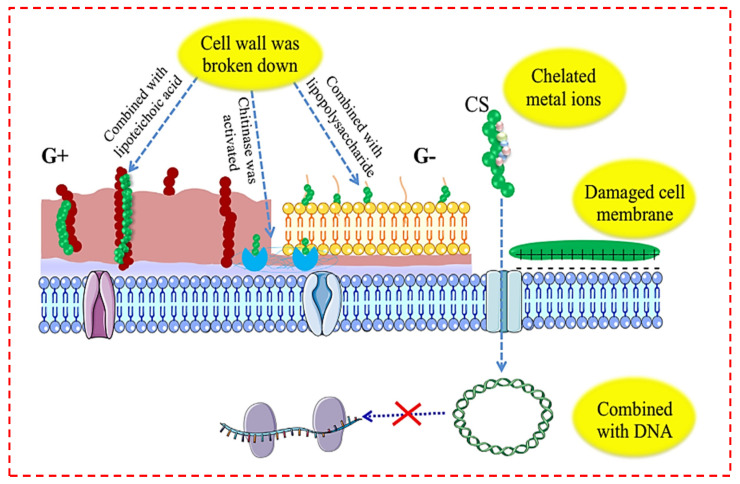
Antibacterial mechanism of chitosan and its derivatives [129].

Chitosan (CS) is regarded to be probably the most preferred antibacterial material because of its natural antibacterial characteristics, extensive source, and high yield [21,128]. The antimicrobial behavior of CS is due to its cationic character. The specific mechanism of the antimicrobial behavior of chitin, CS, and their derivatives is still unidentified, although various mechanisms are offered [107,129]. There are numerous inferences regarding the mechanism of the CS antibacterial effect: (1) interaction between positively charged CS molecules and negatively charged microbial cell walls might result in bacterial biofilm split, and leakage of the resultant proteins and various other cellular elements, which result in bacterial fatality. At lower amounts, binding of positively charged CS to the surface of negatively charged bacteria may result in the accumulation of bacteria; a tremendous number of positive charges might neutralize the charge on the surface of the bacteria and induce the interruption of the bacteria with the escalating CS amount. (2) CS is employed as a chelating agent to selectively bind metal ions, thus suppressing the generation of toxins and the growth of microorganisms. (3) The components of the cell wall membrane are triggered and combined by CS, leading to the bacterial cell wall membrane breaking down and fatality. (4) Chitosan is additionally combined with DNA by infiltrating the nucleus of microorganisms, interfering with and preventing mRNA and protein activity, as shown in Figure 7 [129]. Various antibacterial performances could also imply numerous antibacterial action mechanisms. Primarily, the antibacterial mechanism of powdered chitin and CS was associated with the free amino groups existing in CS, which led to the destruction of the cell wall.

**Figure 8:**
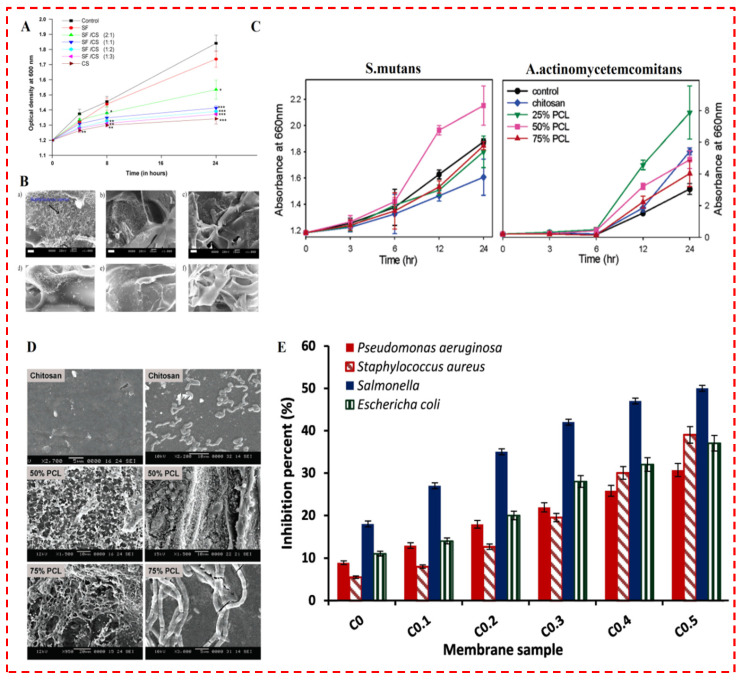
(**A**) Effect of matrix structures and composition on antibacterial activity of SF/CS scaffolds. (**B**) Scanning electron microscopy (SEM) demonstrates attachment of *S. aureus* on neat and blended scaffolds. Neat silk fibroin (SF) scaffolds (**a**), SF/CS (2:1) (**b**), SF/CS (1:1) (**c**), SF/CS (1:2) (**d**), SF/CS (1:3) (**e**), and neat CS scaffolds (**f**). Note: Scale bar = 10 µm [107]. (**C**,**D**) Influence of blending on antibacterial behavior of CS. Chitosan (CS), poly caprolactone (PCL), and blend membranes were soaked in bacterial broths of *S. mutans* and *A. actinomycetemcomitans* and incubated aerobically at 37 °C [108]. (**E**) Antibacterial activity of crosslinked gelatin/CS membranes, with various concentrations of cinnamaldehyde, towards *P. aeruginosa*, *S. aureus*, *Salmonella*, and *E. coli*. Values are portrayed as mean and standard deviation (± SD; n = 3) [109].

The antibacterial behavior of CS has also been evaluated broadly in TE and wound dressing purposes (Table 1). For instance, Zhao et al. [32] produced a new series of in situ-forming antibacterial conductive degradable hydrogels, applying quaternized chitosan- (QCS) grafted polyaniline with oxidized dextran as a crosslinker for TE application. In another study, Bhardwaj et al. [107] presented the porous polyelectrolyte complex scaffolds of silk fibroin (SF) and amino polysaccharide CS. Their group examined the bactericidal and prohibitive capacity of SF/CS blended scaffolds towards bacterial proliferation and attachment. Their examination was carried out utilizing oral pathogen *S. aureus* demonstrated an initial enhancement in optical density (OD) for whole matrices comparable to the control (Figure 8A) [107]. The control comprised only bacterial cell suspension without any scaffolds. Nevertheless, the OD of specimens in suspension diminished from 6 h onwards and was considerably lower compared to those of the control, pure SF and SF/CS (2:1) by 12 h, indicating CS was potent towards the inhibition of *S. aureus* growth and was more efficient than blended scaffolds. SF/CS (1:1), SF/CS (1:2) and SF/CS (1:3) specimens exhibited substantially lower (*p* < 0.001) OD. SEM observation of whole matrices after 24 h revealed a similar trend (Figure 8B) [107]. Chitosan and blended scaffolds with a greater amount of CS demonstrated fewer attachments of *S. aureus* than neat silk and SF/CS (2:1) scaffolds. The antibacterial performance is compromised with escalating silk amounts in the blends [107]. Similar types of findings are also noticed with membranes and blended scaffolds of CS/PCL [108].

In Sarasam et al.’s [108] study, CS and PCL were blended in three various mass ratios (25 wt. %, 50 wt. %, and 75 wt. % of PCL) and processed into membranes. They evaluated the bactericidal properties of CS towards Gram-positive S. mutans by suspending the membrane in bacterial broth and soaking in suitable media. Transient changes in the OD (Figure 8C) [108] of the broth portrayed that, except for 50% PCL, all the biomaterials exhibited a diminished growth of bacteria in suspension than the control sample. There were no significant differences in the OD of the 25% and 75% PCL suspensions. At any given time, the suspensions containing CS membranes demonstrated the least growth of bacteria. Even so, the growth of bacteria in the existence of these membranes pointed out that CS, PCL, or their blends are not bactericidal. When the experiments were repeated with Gram-negative A. actinomycetemcomitans (Figure 8D) [108], growth was detected in suspensions of whole membranes, such as CS, implying that they did not have a bactericidal effect on this pathogen either. Surprisingly, in contrast to S. mutans, the growth rates in the existence of these membranes were greater than in the control sample. This portrayed that the antibacterial performance of CS was more efficient towards S. mutans in comparison with A. actinomycetemcomitans in suspensions. Generally, Sarasam et al. [108] reported that the existence of PCL affected the antibacterial characteristics of CS [108]. In some studies, drug release is also utilized to enhance the antibacterial impact of CS, as conducted by Kenawy et al. [109]. In their study, examination wound dressing was fabricated from a CS biopolymer. Their study aims to prepare absorbable and antimicrobial membranes depending on crosslinked gelatin/CS biopolymers. Furthermore, cinnamaldehyde was encapsulated in the membranes to elevate their antimicrobial actions. Their results demonstrated a substantial enhancement in the inhibition percent with an escalating cinnamaldehyde amount in the membrane matrix [109]. Figure 8E depicts the antimicrobial performance of the developed crosslinked membranes towards three Gram-negative bacteria (*P. aeruginosa, Salmonella, and E. coli*) and one Gram-positive bacteria (*S. aureus*) [109]. A substantial escalation in the inhibition activity was detected with escalating cinnamaldehyde content. This enhancement can be described through Schiff base creation, where coupling of amine groups with the aromatic aldehyde groups of cinnamaldehyde might create hydrophobic sides of a long membrane matrix. This consequently increases the interaction with peptidoglycan in the cell wall and lipoprotein in the outer membrane layer and simplifies its adhesion to the microorganism cell wall membrane. In addition, the inhibition (%) towards Gram-negative bacteria was greater than Gram-positive bacteria. The interaction of positively charged amine groups with the negatively charged cell surface leads to substantial modifications of cell wall membrane permeability [109].

## 4. Printed Constructs from Antibacterial Inks

Three-dimensional (3D) printing offers controlled deposition and patterning of polymeric or composite biomaterials, to produce well-defined constructs with the ability to combine various materials and their compositions. 3D printing and bioprinting techniques are progressively used for the biofabrication of 3D constructs in tissue engineering (TE). The 3D printing of biomaterials, termed biomaterial inks, allows the deposition of biomaterials in a well-defined 3D scaffold, which can develop into living tissue-engineered constructs. 3D bioprinting offers admission to well-defined structures, accomplishing a varied range of mechanical and biological properties. Nevertheless, the accessible biomaterial inks and bioinks, which present proper printability and favorable antibacterial performance for bioprinting and 3D printing, remain limited [22]. Bioprinting is achievable via various 3D printers, which are more and more reasonable, consistent, and user friendly, extending from elementary to sophisticated setups [23].

### 4.1. Metallic Ion-Containing Inks

Synthetic polymers are extensively utilized for bone TE because of their tunable physical characteristics and biocompatibility. Naturally, most of these polymers exhibited poor antimicrobial behavior. Infection at a position close to the implantation site is a key factor for failure or postponement in the bone healing procedure, and the production of antimicrobial polymers is preferred. In this respect, silver has been the most commonly utilized antibacterial agent for generating antibacterial inks in recent years, as summarized in Table 2.

**Figure 9:**
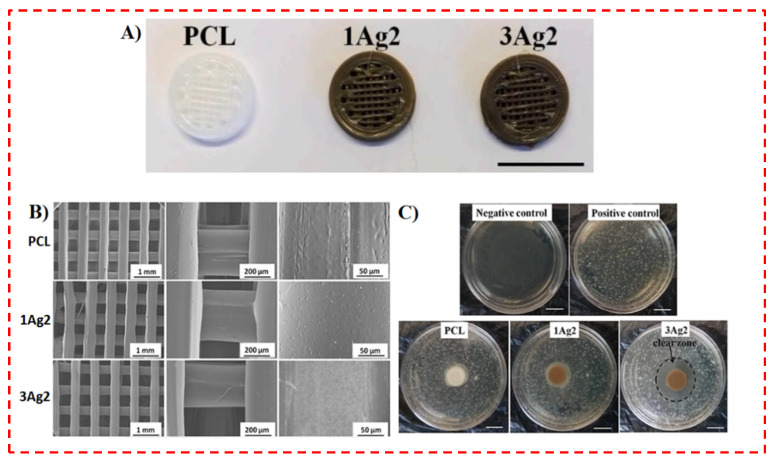
(**A**) Photograph of the 3D printed scaffolds (scale bar denotes 1 cm), (**B**) SEM image of 3D printed scaffolds, and (**C**) Soft agar plates inoculated with *E. coli* cells and incubated with 3D printed scaffolds in their center for 24 h (Scale bar denotes 1 cm) [37].

Radhakrishnan et al. [37] prepared AgNPs-PCL solution by in situ reduction. In detail, PCL/AgNPs composites were synthesized utilizing 1% and 3% AgNO_3_ via a customized 3D printing technique, which are labeled as C1Ag2 and C3Ag2, respectively (Figure 9A). PCL, C1Ag2, and C3Ag2 were extruded into filaments; additionally, they prepared 3D structures utilizing the PCL/AgNPs filaments via an FDM-based 3D printer (PRUSA i3) (Figure 9B) [37]. Figure 9 depicts the results attained for their 3D printed scaffolds from an inhibition examination conducted at 24 h. Negative control plates (without bacteria) revealed clear nutritive soft agar plates. However, positive control plates (with bacteria) demonstrated a homogeneous distribution of bacterial colonies inside the soft agar (Figure 9C) [37]. With the 3Ag2 scaffold, a clear IZ (20.4 ± 1.7 mm) was detected in contrast to no obvious IZ being detected for PCL and 1Ag2, as shown in Figure 9C. The existence of an IZ around the 3Ag2 scaffolds evidences an antibacterial performance depending on the infiltration of biocidal agents employing the soft agar. These biocidal agents could be silver (i.e., Ag0 instead of Ag^+^ based on XPS analysis) and/or ROS [37].

Another recently published research paper on 3D printed constructs encapsulated with antibacterial metallic ions is reported by Afghah et al. [36], who prepared a 3D printable copolymer based on polycaprolactone-block-poly(1,3-propylene succinate) (PCL-PPSu), loaded with antimicrobial Ag particles for STE. In their study [36], the *E. coli, P. aeruginosa, S. aureus,* and *C. albicans* pathogens, which are associated with infections detected in implants or burn wound areas, were seeded on lysogeny broth (LB) agar Petri dishes by spreading from overnight cultures. Subsequently, the copolymer films with/without AgNO_3_ were positioned meticulously on the LB agar. After overnight incubation, no indication of antimicrobial influence towards *E. coli, P. aeruginosa, S. aureus*, and *C. albicans* strains was observed in the copolymer specimens. Nevertheless, copolymers encapsulated with AgNO_3_ displayed antimicrobial behavior towards all the tested microorganisms. Obvious IZs were experienced close to the copolymer films, with varying sizes based on the various microorganisms. Moreover, PCL-PPSu/AgNO_3_ films exhibited positive antimicrobial performance versus C. albicans, a harmless member of the human microbiome, which might result in life-threatening infections within particular circumstances [36]. In a similar fashion, Li et al. [133] manufactured PCL scaffolds by FDM incorporated with polydopamine (PDA) and Ag as an antibacterial agent. They reported no significant IZ for *S. aureus* was experienced for PCL scaffolds and PCL/PDA scaffolds throughout 14 days of in vitro examination. The IZ for *S. aureus* utilizing PCL/AgNPs scaffolds lessens to zero after 14 days of culture. Employing PCL/PDA/AgNPs scaffolds, the IZ value presented a progressive reduction with a sustainable antibiotic release after 14 days’ culture, where the role of Ag as an antibacterial agent is obvious [133].

**Figure 10:**
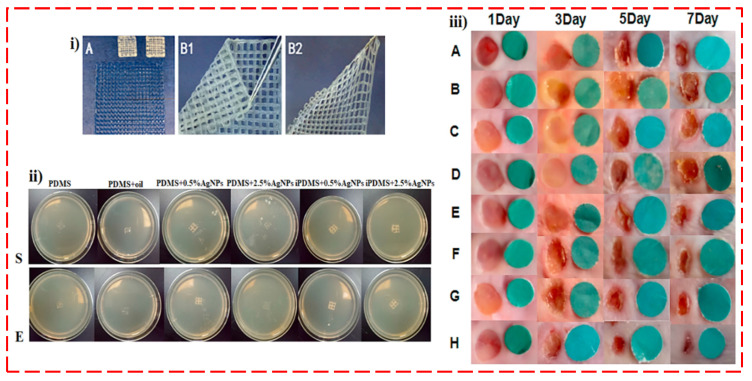
(**i**) Micrographs of 3D-printed mesh and nanosilver dotting. A: iPDMS membrane with flexibility and different sizes; B1: folded oil-infused 3D-printed polydimethylsiloxane with antibacterial nanosilver (iPDMS/AgNPs), and B2: stretched PDMS/AgNPs. (**ii**) The gross appearance of bacteria in the six groups (co-cultured with *S. aureus* or *E. coli*). (**iii**) The wound profiles of the infected wound from 1 day to 7 days [135]. Note: Negative control (**A**), positive Control (**B**), PDMS (**C**), PDMS + oil (**D**), PDMS + 0.5%AgNPs (**E**), PDMS + 2.5%AgNPs (**F**), PDMS + 0.5%AgNPs + oil (**G**), and PDMS+2.5%AgNPs+oil (**H**).

In addition to TE, antibacterial agents are one of the essential aspects of wound healing processes. For instance, the Ag-ethylene interaction and the 3D printing approach employed by Wu et al. [134] to create the antibacterial super-porous polyacrylamide (PAM)/hydroxypropyl methylcellulose (HPMC) hydrogel dressings via a home-made 3D bioprinter. They revealed that Ag-ethylene interaction is responsible for mediating the creation, distribution, and crosslinking of AgNPs in the hydrogel matrix, along with the crosslinking of the PAM networks. Simultaneously, this kind of organometallic complex additionally manipulated the generation of AgNPs to balance the cytocompatibility and antibacterial property of the AgNP-crosslinked hydrogels [134]. Other polymeric inks encapsulated with Ag ions for wound dressing purposes were presented by Shi et al. [135] (Table 2). As can be observed, iPDMS/AgNP is effortlessly folded and stretched (Figure 10i) [135]. Their findings depicted that the oil-infused 3D printed polydimethylsiloxane, with antibacterial nanosilver (iPDMS/AgNPs) at 0.5 wt. % and 2.5 wt. %, can cater to the various specifications of wounds, with antifouling, anti-blood staining, and can kill bacteria (Figure 10ii) [135]. Moreover, iPDMS/AgNPs not only displayed biocompatibility and excellent antibacterial behavior, but likewise successfully elevated neo-epithelial and granulation tissue creation to boost the wound healing rate (Figure 10iii) [135].

Ag_3_PO_4_ and lidocaine-encapsulated PCL scaffolds were printed utilizing pneumatic extrusion-based 3D printing in Shao et al.’s [138] study. They exhibited the antibacterial characteristics of Ag-encapsulated inks. The released medium from their Ag-encapsulated scaffolds demonstrated a noticeable IZ towards *S.aureus* and *E.coli* upon encapsulation with 1% Ag_3_PO_4_ for up to 6 days and with 3% Ag_3_PO_4_ for at least 7 days. In terms of cytotoxicity evaluation, they reported that although the Ag_3_L_4_ scaffolds (encapsulated with Ag and lidocaine) revealed a toxic influence to MC3T3 cells, which could be owing to the hypertonic influences of the dual-released lidocaine and Ag, the solely lidocaine- or Ag_3_PO_4_-encapsulated scaffolds did not stimulate a toxicity effect for MC3T3 cells [138].

**Figure 11:**
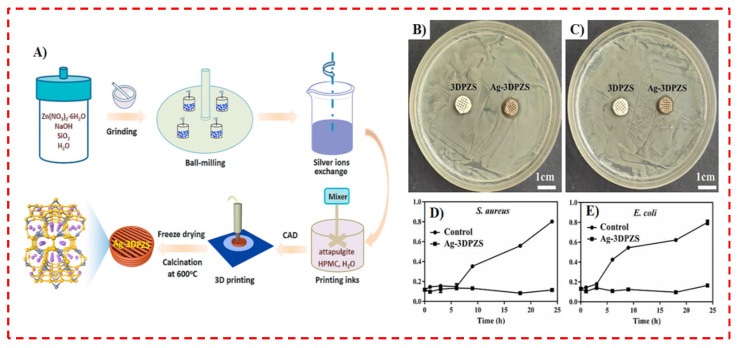
(**A**) Schematic illustration of overall fabrication process of Ag-3DPZS via 3D printing and representative photographs of the bacterial inhabitation zone (IZ) created by the different scaffolds against *S. aureus* (**B**) and *E. coli* (**C**); bacterial growth kinetics in liquid medium: *S. aureus* (**D**) and *E. coli* (**E**) [131].

In addition to encapsulating silver into polymers, incorporating this ion into ceramics is another well-known approach for generating antibacterial inks, as was investigated in Wang et al.’s [131] study. Wang et al. [131] encapsulated silver in 3D printing inks to attain antibacterial constructs for BTE purposes. They manufactured Ag-encapsulated zincosilicate zeolite scaffolds (with zeolite Ag-VPI-7 powders as the primary raw materials) through the extrusion 3D printer, as illustrated in Figure 11A [131]. In the printing approach, they employed the nanofibrous attapulgites, with appropriate rheological characteristics and great bioactivities, as inorganic binders, which elevated the compressive strength and Young’s modulus of Ag-3DPZS up to 8.38 and 111 MPa, respectively, where it is mentioned that the mechanical properties are crucial for BTE purposes [25,26,131]. Figure 11B,C show obvious bacterial IZs on the agar plate generated by Ag-3DPZS (the right scaffolds in the figure), while there are no IZs around the 3D-printed zeolite scaffolds without the encapsulation of Ag ions (3DPZS, the left scaffolds in the figure) [131]. The outcomes demonstrate that Ag-3DPZS has an inhibitory influence on both bacteria (*S. aureus* and *E. coli*) caused by the Ag release into the agar medium. Nevertheless, 3DPZS shows no bacteriostatic performance. The bacteria growth trends related to *S. aureus* and *E. coli* presented in Figure 11D,E exhibited that Ag-3DPZS drastically prevents the growth of *S. aureus* and *E. coli* at each test point, implying the broad-spectrum antibacterial behavior of Ag-3DPZS [131]. Since Ag-3DPZS was employed as an antibacterial scaffold for BTE, it was required to assess its cytotoxicity. In this context, Wang et al. [131] incubated the MC3T3-E1 cells for 48 h with the leaching solutions of various specimens; there was no significant difference between the Ag-3DPZS specimen and the control specimen, implying the non-cytotoxicity of the Ag-encapsulated zeolite scaffold. Ultimately, they presented Ag-3DPZS as an antibacterial and cytocompatible scaffold for BTE, with excellent mechanical behavior [131].

Another examination on incorporating Ag ions to attain antibacterial inks regarding bone reconstruction is Zhang et al.’s [132] study. In their study, AgNPs were evenly dispersed on GO to create a homogeneous Ag@GO coating with various Ag-to-GO mass ratios, with this being fabricated via the liquid chemical reduction approach. Ag@GO nanocomposites were effectively treated on the 3D-printed β-TCP scaffolds simply by a soaking approach to attain bifunctional biomaterials with antibacterial and osteogenic action. They revealed that the scaffolds with Ag@GO coating displayed remarkable antibacterial behavior towards *E. coli,* as well as enhanced ALP and enhanced osteogenic differentiation of rabbit bone marrow stromal cells (rBMSCs) [132]. Zinc oxide is another antibacterial agent employed by Zhu et al. [136], which displayed 3D-printed ceramic hip joints with accurate structures and excellent antibacterial performances implementing ZnO-layered treated ZrO_2_. Their result depicted that the ZrO_2_-ZnO ceramics had an excellent antibacterial performance versus *E. coli* and *S. aureus* within 8 h, and above 80% viability of MC3T3-E1 cells for all specimens [136]. Copper has been exhibited to have a successful antibacterial performance towards a variety of bacteria, such as *S. aureus* [139] and *E. coli* [140], the most prevalent microorganisms in bone infections. Hence, it seems that the encapsulation of Cu into 3D printing inks is usually important for TE purposes. In this regard, Cu-encapsulated zeolitic imidazolate-framework (ZIF-8) nanoparticles and poly (lactide-co-glycolide) (PLGA) were combined to prepare PLGA/Cu(I)@ZIF-8 scaffolds, utilizing 3D printing approaches for infected bone repair in Zou et al.’s [137] study. They revealed that in vitro, *S. aureus* cultured on the PLGA/Cu(I)@ZIF-8 scaffolds were practically all dead, while in vivo inflammatory cell infiltration and bacteria numbers were considerably diminished in infected rats implanted with PLGA/Cu@ZIF-8 scaffolds. In addition, the mMSCs cultured on the surface of PLGA/Cu(I)@ZIF-8 scaffolds were attached, and spread, and staining with ALP and alizarin red were more amplified than with neat PLGA scaffolds. The mineralization assay demonstrated an apatite-rich film was created on the surface of PLGA/Cu(I)@ZIF-8 scaffolds, while barely any apatite was detected on the surface of the PLGA scaffolds [137].

### 4.2. Chitosan-Containing Inks

**Figure 12:**
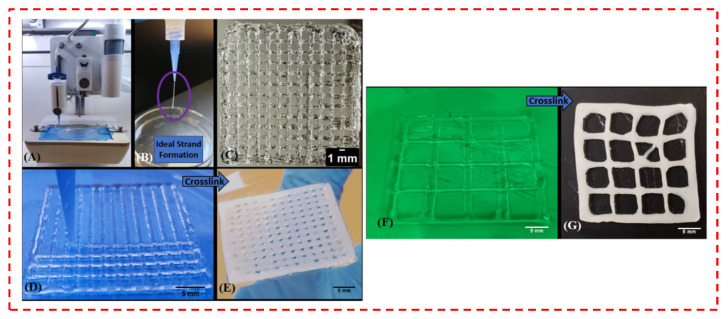
Bioprinting of scaffolds using chitosan (CS)-polyethylene oxide-glycerol phosphate (CPG) gel. (**A**) The bioprinting system, consisting of a temperature-controlled printhead and printbed. (**B**) A printed structure showing the ability of the gel to form ideal. (**C**) Printed scaffold with square pores. Scaffolds (**D**,**F**) before thermal gelation, and (**E**,**G**) after thermal crosslinking at 37 °C for approximately 770 s. Scale bars = 5 mm [130].

The printing of CS ink has actually been examined for around 10 years via implementing certain factors, including printing in Petri dishes comprising dry ice [141,142]. Nevertheless, few reports exist regarding the antibacterial performance of CS inks, and productive examination regarding boosting this crucial feature for protecting against the resultant infections of bone scaffold implantation has been ignored. Tardajos et al. [39] employed CS in order to fabricate antibacterial inks. They established an innovative surface treatment approach on electrospun and 3D-printed PCL scaffolds by grafting methacrylic acid N-hydroxysuccinimide ester (NHSMA) onto the surface following Ar-plasma/air activation, to subsequently react the introduced NHS groups with different molecular weights (Mw) of CS as potential antibacterial materials. The antibacterial assessment towards *S. aureus* and *S. epidermidis* pointed out a sluggish bacterial growth rate on the surface of the CS-treated scaffolds, independent of the Mw of the chitosan. Furthermore, a lactate dehydrogenase assay (LDH) employing L929 fibroblasts exhibited the cell adhesion and cell viability potential of the treated samples [39]. In spite of the remarkable development in the improvement of scaffolds that can enhance bone healing, bacterial colonization of surgically implanted scaffolds continues to be a clinical issue that could endanger the results of the regenerative treatment [143]. In this respect, Ramesh et al. [130] presented the formulation and subsequent 3D deposition of a material system mixing the osteoconductivity of CS-glycerol phosphate (GP) hydrogel with the antibacterial performance of ZnONPs for purposes in BTE [130]. Nanoparticles presenting antibacterial properties attract huge attention in scaffold-based TE techniques, caused by their simplicity of encapsulation and for the manufacturing of well-designed 3D scaffolds. Ramesh et al. [130] fabricated 3D scaffolds of CS-polyethylene oxide (PED)-glycerol phosphate (CPG) using a bioprinter in a controlled atmosphere, and CPG scaffolds before and after crosslinking are shown in Figure 12 [130]. Likewise, the influence of the ZnO amount on antibacterial behavior was analyzed utilizing UV-treated ZnONPs at different amounts, namely 0.01, 0.05, 0.1, 0.5, 1, 2, and 4 mg/mL. To identify the NPs’ characteristics with the highest antibacterial performance, ZnONPs of sizes 20 and 90 nm, both with (UV+) or without (UV-) being exposed to UV light, were examined for their antibacterial performance towards *E. coli*. ZnONPs of 90 nm size treated with UV demonstrated the greatest antibacterial performance, with nearly a 4-log reduction in bacterial survival as opposed to the control (without ZnO). ZnO at a higher amount (2 mg/mL) led to the prevention of more than 99.99% of the growth for *E. coli*; however, even 1 mg/mL drastically prevented the growth of *S. aureus*, with inhibition of 99.999% of the cell. Gram-positive bacteria have typically been detected to be more sensitive to ROS-mediated killing [144], which might reveal the greater antibacterial performance of ZnONPs towards the Gram-positive *S. aureus* than that of the Gram-negative *E. coli*.

### 4.3. Other Antibacterial Inks

Li et al. [53] prepared a PLA scaffold through the FDM approach that consisted of extremely interconnected porosity, adequate nutrient supply, and antibacterial performance. Accompanied by dopamine polymerization on the surface of the substrate, grafting with gelatin (Gel)/hydroxyapatite (HA) and ponericin G1 was additionally performed. In their examination, both *E. coli* and *S. aureus* were significantly prevented up to 24 h, and the IZ could remain for 72 h. Specifically, when the amount of ponericin solution was controlled at 250 μg/mL, the antibacterial performance did not differ with the raised amount of gelatin, implying that the integrating ponericin amount is not dependent on the viscosity of the solution. Nevertheless, when the amount of gelatin was controlled at 3%, the OD value of the bacteria culture was diverse to a significant level in proportion with the amount of ponericin solution (ranging from 50 to 750 μg/mL). For *E. coli*, the greatest antibacterial performance was accomplished at 250 μg/mL, although for S. aureus, the value amplified to 500 μg/mL. After 24 h culture, the identical trend was attained both for *E. coli* and *S. aureus* as at 18 h, and the comparative concentration was still fulfilled with merely a small reduction in the antibacterial behavior. Their findings demonstrated that the ponericin encapsulated scaffold was more sensitive to *E. coli* compared with *S. aureus* and the scaffold was capable of maintaining a long-term antibacterial behavior [53].

## 5. Conclusions and Future Perspectives

Biomaterials perform an essential function in disease treatment; however, the presence of infective microorganisms on biomaterial surfaces lead to major issues that seriously restrict the functional use of these devices. To create biomaterials with anti-infective properties, a number of approaches have been developed. The numerous antibacterial materials that have been intrinsically used for biomedical applications include metals (e.g., Ag, Zn, and Cu), polymeric materials (e.g., CS) and their composites, and ceramics (e.g., ZnO, MgO, and TiO_2_). Due to their intrinsic antibactericidal activity, these materials have been extensively employed in numerous biomedical applications.

Biomaterial inks and bioinks with characteristic antibacterial activity are of specific attention for TE application due to the increasing number of bacterial infections accompanied by the compromised regeneration of tissues. Nevertheless, the achievement of cell-laden bioink by effective antibacterial activity, although associated with tissue regeneration, is demonstrated to be challenging [24]. In order to address this problem, novel antibacterial bioinks have been established for 3D bioprinting in TE applications.

Over the past decades, 3D printing techniques have been rapidly developed and widely employed to create scaffolds for various TE applications. Nevertheless, many issues remain to be addressed for the applications of 3D-printed biomaterials, such as regulatory concerns, a sterile environment for component manufacturing, and the accomplishment of target material characteristics with the preferred structures [25]. For creating efficient TE constructs, multi-material structures consisting of cells might be printed along with organic or inorganic components. These strategies have been drawing extensive attention, while leaving a lot to be desired. Among them, cell viability is a crucial issue for the bioprinting process and the shelf-life of materials, with and without cells, is another considerable challenge. Furthermore, due to considerable variation in the stiffness of the cells and materials to be deposited, careful procedure optimization is required to create scaffolds that could be employed for additional in vitro or in vivo examination. Another essential requirement is vascularization or new blood vessel network creation, based on the principles of angiogenesis and vasculogenesis [145]. Alternatively, the escalating infectious diseases after operations are primary restrictions in biomedical applications [146]. Bacterial infections on the surface of the biomaterial biofilm have threatened to utilize biomaterials in the body [146]. Regardless of the reliable host immune system, the implant surface might be quickly filled by bacteria, leading to infection persistence, implant failure, and even death of the patients. It is challenging to deal with these issues due to the fact that bacteria display complex attachment mechanisms to the implants that differ based on bacterial strains. Various biomaterial coatings have been created to generate antibiotics to destroy bacteria. Nevertheless, antibiotic resistance takes place very regularly. Hence, encapsulating antibacterial agents into the biomaterial matrix has attained much consideration in the past few years [147]. Antibacterial biomaterials are comparatively capable of repelling bacterial cells, inhibiting their adhesion, or inactivating/destroying cells attached to the surface, while not sufficiently productive in destroying the pathogens due to the complicated mechanisms of bacteria [148,149,150,151,152,153,154,155,156,157,158,159,160,161,162,163,164,165,166,167,168,169,170,171,172]. Thus, developing highly effective and specifically targeted biomaterials that generate antimicrobial agents and their service in 3D printing inks is vital for TE applications. In addition, adopting the mechanisms for preventing bacterial attachment and biofilm creation is crucial. In this regard, the majority of research for presenting favorable inks containing antibacterial features, including metallic and metal oxide (e.g., Ag, Cu, Au, TiO_2_, MgO, and ZnO) nanoparticles with low cytotoxicity, is potentially going to be broadly employed in the foreseeable future for eliminating numerous infectious situations. Regarding antibacterial inks based on natural polymers such as CS, numerous essential factors, including the difficulties of print parameter optimization, selecting suitable and non-toxic crosslinkers which do not clog the nozzle, and issues of structure stability preservation, should be considered before the bioprinting tissues. In addition, more significant attention should be provided regarding the interactions between polymers and metallic ions in order to fabricate well-designed tissue constructs with high antibacterial performance. Furthermore, other aspects, including evaluating the biocompatibility of the antibacterial inks encapsulated with metallic ions and obvious concentration on the non-harmful limit to biocompatibility, likewise necessitate extensive exploration. The latest advancements in approaches for fabricating antibacterial inks in combination with great printability could be very valuable for the manufacturing of innovative bactericidal biomaterials, for the preservation of their long-term performance, and aid in reducing biomaterial infection in the body. Taken together, we believe that bioink-related biomaterials containing antibacterial agents are developing very rapidly and pave the way for further research to prevent infection during/after implantation.

## Figures and Tables

**Table 1:** Utilization of materials with antibacterial properties in biomedical applications.

Materials	Antibacterial Nanoparticles	Structure	Application	Results	Ref.
HAp-PEG	Cu	NP	BTE	The antibacterial performance of nHA-Cu/PEG specimens was higher, and they were more effective toward Gram-positive pathogens than Gram-negative strains.	[49]
CS-PEG	Cu	Microporous hydrogels	Wound dressing	The addition of Cu^2+^ to the CS-PEG films escalated the films’ antibacterial performance.	[50]
Silicate MBG-Pluronic P123	Cu	Powder	BTE	The concentration of Cu in the MBG composition influenced both structural and functional characteristics: as Cu levels grew, SSA dropped, but antibacterial performance towards *S. aureus* escalated.	[51]
Wollastonite	Cu	Particles	BTE	The incorporation of Cu to the wollastonite improves the inhibition zone against both *S. aureus* and *E. coli* strains; however, the growth inhibition towards Gram-positive bacteria strains was determined to be extremely effective.	[52]
HAp	Cu	Scaffold	BTE	Cu was added to the HA scaffolds, which escalated antimicrobial performance. On day 7, the cells on the 5Cu–HA scaffolds treated with a 5% CuSO_4_ curing solution showed good growth.	[53]
BG	Cu	Scaffold	BTE	The scaffolds escalated cell response, including cell viability and cell attachment, drug delivery and antibacterial behavior.	[54]
PGF	Cu	Fiber	Wound healing	The opportunistic bacterium S. epidermidis was killed most effectively by the Cu^2+^ ions produced by the 10% CuO glass fibers.	[55]
ESM-BG	Cu	Membrane	Wound healing	The 5Cu-BG/ESM films were able to generate Cu^2+^ ions for an extended period of time and effectively suppressed the survival of bacteria (*E. coli*). Cu^2+^ ions produced by Cu-BG/ESM nanocomposite films have a critical role in angiogenesis and antibacterial behavior.	[56]
BG, PCL	Cu	Coatings	Coating for Mg-based biomaterials	The generation of Cu^2+^ ions from Cu-BGN coatings inhibited the growth of *S. carnosus* and *E. coli*.	[57]
CPS	Cu	Powder	BTE	After sintering at 1200 °C, the bending strength of CPS increased from 29.2 MPa to 63.4 MPa with the addition of 3.0 wt. % CuO. Cu-CPS bioceramics outperformed *S*. *aureus* and *E. coli* strains in vitro, demonstrating greater antibacterial performance.	[58]
GO	Cu-Ag	Powder	Biomedical	GO/AgNPs and GO/CuONPs presented significant antibacterial performance.	[59]
CS-HAp	Cu-Zn	Scaffold	BTE	The incorporation of nCu-Zn to the CS/nHA scaffolds boosted swelling, reduced breakdown, escalated protein adsorption, and enhanced antibacterial behavior, while causing no toxicity in rat osteoprogenitor cells.	[60]
PCL	Ag	Membrane	Wound dressing	Up to 0.5 wt. % AgNPs concentration, tensile strength, elongation at break, and tensile modulus were substantially greater for PCL/Ag nanocomposite membranes. After incorporating 1 wt. % AgNPs, the PCL’s intrinsic elastic nature transformed to a brittle nature. The antibacterial performance of PCL/Ag toward *S*. *aureus* and *E. coli* was outstanding.	[61]
HAp/Gel/Alg/PVA	Ag	Scaffold	BTE	The nanocomposite scaffolds exhibit compressive strength in the range of 4.02 to 29.5 MPa and modulus in the range of 34 to 198 MPa, according to their mechanical characteristics. The scaffolds have a great antibacterial performance toward *Bacillus* and *E. coli*.	[62]
CS-PEO	Ag	Nanofibers	Wound dressing	The incorporation of Ag to the CS/PEO blend solutions improved the mechanical performance of the CS/PEO nanofiber mats. The antibacterial test revealed that Ag-CS/PEO nanofiber mats exhibited good bactericidal behavior toward both Gram-negative *E. coli* and Gram-positive *S. aureus* bacteria.	[63]
PLA	Ag	Nanocomposite	TE	With an escalation in the concentration of AgNPs in the PLA, Ag/PLA-NC films had a considerable antibacterial performance.	[64]
Cellulose/ PANI	Ag	Aerogels	STE	The antibacterial performance of BC/Ag/PANI aerogels toward *E. coli* and *S. aureus* bacteria was substantial.	[65]
CS	Ag	Scaffold	BTE	Antimicrobial performance, biocompatibility with mammalian cells, and enhancement of osteogenic differentiation were observed in the CS-Ag scaffold.	[66]
HA	Ag	Matrix	TE	AgNPs and HA/SNPs, unlike neat HA, displayed antimicrobial action	[67]
SF	Ag	Scaffold	BTE	The antibacterial performance of silk fibroin films encapsulated with AgNPs was tested toward both Gram-negative and antibiotic resistant bacteria, and it was observed to be successful in both cases.	[68]
Starch/PVA	Ag	Nanofibers	STE	The antimicrobial property was enhanced by coating the nanofibers with AgNPs	[69]
HAp	Ag	Nanopowders	BTE	In vitro antibacterial behavior of Ag-doped hydroxyapatite specimens toward *S. aureus*, *E. coli*, and Candida albicans pathogens has been reported in antimicrobial experiments.	[70]
Mg	Ag	Scaffold	BTE	The antimicrobial behavior of Mg-based scaffolds encapsulated with Ag was examined, and it was observed that escalating the content of incorporated Ag suppressed the development of *E. coli* and *S. aureus* in the IZ around the Mg-based scaffolds.	[71]
CS/PU	Ag	Membrane	DBM and TE	The AgNPs in the membrane were found to have an antimicrobial impact. A medical dressing membrane fabricated from a CS membrane incorporating a trace concentration of AgNPs can be employed.	[72]
CS	Ag	Scaffold	Skin TE	Ag was responsible for the Ag@CMs/CS scaffold’s good antibacterial behavior owing to its prolonged release of Ag@CMs. Nevertheless, all Ag@CMs/CS scaffolds demonstrated good cell growth and spread, as well as an escalation in antibacterial activity, owing to their sustained release features.	[73]
Alg/HAp	Ag	Scaffold	BTE	Silver has been shown to have no influence on the scaffolds’ ability to enhance osteoblast proliferation, while also having a significant bactericidal effect toward both Gram-positive and Gram-negative bacterial strains in in vitro biological studies.	[74]
CS/HAp	Ag	Scaffold	BTE	The IZ of the CS/nHAp/nAg scaffolds toward *E. coli* and *S. aureus* was determined to be 13.34 ± 2.75 mm and 12.78 ± 1.10 mm, respectively.	[75]
PHBV	Ag	Scaffold	TE	Only silver incorporating PHBV nanofibrous scaffolds had significant antibacterial performance and inhibited the growth of *S. aureus* and *K. pneumoniae* bacteria.	[76]
Gel/PCL	Ag	Scaffold	TE	Except for the Ag-coated PCL nanofibrous scaffold loaded with 1.25% AgNO_3_ solution, there was an obvious IZ around Ag-coated nanofibrous scaffolds for both Gram-positive and Gram-negative bacteria. Only 0.8% Ag was detected in this specimen. The bacteria tested were not destroyed by the low dose of Ag. Antimicrobial effects were detected when the Ag amount was escalated to 4.2%.	[77]
PCL	Ag	Scaffold	TE	AgNPs escalated the antibacterial behavior of PCL scaffolds, according to disc diffusion experiments.	[78]
SF/HAp	Au-Ag	Hydrogels	BTE	Both Gram-positive and Gram-negative bacteria were inhibited significantly by hydrogels containing AgNPs and AuNPs. Utilizing osteoblastic cells, cytocompatibility experiments demonstrated that the hydrogels can be employed as antimicrobial materials with up to 0.5 wt. % AgNPs and all amount of AuNPs, without impairing cell behavior.	[79]
DEG	Au-Ag	Hydrogel	Wound healing	Antibacterial activity of Ag encapsulated hydrogels has been found to be greater compared to Au encapsulated hydrogels.	[80]
CS	Au-Ag	Nanocomposites	Wound dressings	In vivo results exhibited that CS-Au-Ag enhanced wound healing significantly more than CS-Ag, indicating that CS-Au-Ag has considerable potential as a wound dressing.	[81]
CS/PVA/HAp	Au-GO	Film	BTE	In all experiments, the IZ for the CS/PVA/HA/Au composite film was greater than for the CS/PVA/HA film. Moreover, the Cs/PVA/GO/HA/Au film presented the highest antibacterial performance.	[82]
PMMA	Au	Bone cement	TKA, THR	In comparison to control specimens (without AuNPs), live bacterial cells were diminished by up to 54% and 56% for MRSA and Pseudomonas, respectively, on bone cements made by incorporating 1 wt. % AuNPs.	[83]
CS/PVA	Au	NP	Wound healing	For the lowest and highest encapsulation of AuNPs, the IZs grew from 4.2 ± 0.9 mm to 13.1 ± 1.3 mm versus *E. coli* and from 6.4 ± 1.2 mm to 24.8 ± 2.4 mm versus *S. aureus*, respectively.	[84]
PCL/Gel	APA-coated Au	Scaffold	Wound dressings	Even when exposed with MDR bacteria, APA-treated AuNPs (Au-APA) showed significant antibacterial performance. It also exhibited a remarkable capacity to treat MDR bacteria wound infections.	[85]
CS	Au Nanoclusters	Nanoaggregate	Wound healing	In contrast to their individual components, the synergetic combination fabricated by the Au and CS in the nanoaggregates led to a greater antibacterial action versus *E. coli* and *S. aureus* bacterial strains.	[86]
Gold	Au	NP	Wound healing	Many conventional antibiotics have lower antibacterial and antifungal activities than AuNPs@F. vulgaris. AuNPs@F. vulgaris also inhibited all bacteria from growing at 28 mg/mL concentrations and completely eradicated them at 216 mg/mL concentrations.	[87]
Gold	Au	NP	Wound healing	GNPs generated by *H. hemerocallidea* had an antibacterial effect versus all of the microorganisms examined; however, GNPs generated by G. africana had an antibacterial effect solely versus Pseudomonas aeruginosa.	[88]
PEG	Au	Hydrogel	Wound healing	PEG-AuNRs and PAH-AuNRs hydrogels showed significant antibacterial behavior in vitro versus *S. aureus* and *P. aeruginosa*, as well as great tissue regeneration characteristics when applied topically to wounds in an animal model.	[89]
SA/Cellulose	ZnO	Fibers	Biomedical	The antibacterial performance of the effectively manufactured ZnO-SA-cellulose nanofiber versus *E. coli* was outstanding.	[90]
Col	ZnO	Nanocomposites	Wound healing	In the existence of all Col-ZnO wound dressings, the development of *S. aureus* strains was suppressed. Nanostructured wound dressings have a 5 mm growth zone of inhibition.	[91]
CS/Gel	ZnO	Scaffold	STE	While CS has antibacterial characteristics, its antimicrobial effects are inhibited at neutral pH. The antibacterial behavior of the scaffolds was raised as the ZnO content was escalated.	[92]
Alg	ZnO	Nanocomposites	Medical	After 2 h of exposure, all of the ZnO–alginate nanocomposite specimens demonstrated fast and significant antibacterial action, with a 99.9% decrease for *S. aureus* and a 100% decrease for *E. coli*.	[93]
GO-COOH	ZnO	Nanocomposites	BTE	Against *S. mutans*, ZnO/GO-COOH nanocomposites demonstrated an antibacterial activity.	[94]
SA/PVA	ZnO	Nanofibers	Wound dressing	The antimicrobial effect of SA/PVA/ZnO mats was tested using two bacteria strains: S. aureus and *E. coli*, and it was revealed that SA/PVA/ZnO mats have an antibacterial effect owing to ZnO nanoparticles.	[95]
CMC	ZnO	Hydrogel	Biomedical	Antibacterial characteristics are better in hydrogels containing more ZnO nanoparticles. Gram-positive bacteria were more resistant to CMC/ZnO nanocomposite hydrogels compared to the Gram-negative bacteria.	[96]
PU	ZnO-fMWCNTs	Scaffold	BTE	Electrospun scaffolds comprising 0.2 wt. % ZnO and 0.4 wt. % fMWCNTs were shown to have an antibacterial effect and good biocompatibility, as well as unique bioactive characteristics and cell–biomaterial interaction.	[97]
Al-doped ZnO (AZO)	ZnO-Al	NP	Biomedical	Al-doped ZnO (AZO) zone of inhibition versus *E. coli* and *E. hirae* was reported to be 10.19 ± 0.04 mm and 10.20 ± 0.02 mm, respectively. Electrostatic interactions influenced the antibacterial behavior of AZO, which was reported to be escalated when compared to ZnO.	[98]
PCL/HAp	ZnO	Scaffold	BTE	An antibacterial effect was seen in all PCL:ZnO scaffolds versus *S. aureus*, which could be related to the generation of Zn^2+^ ions.	[99]
PCL	ZnO	Nanocomposites	TE	Pure PCL membranes and fiber mats with less than 5% ZnONPs exhibited less significant action toward the germs tested. The PCL membrane encapsulated with 5% ZnONPs demonstrated statistically significant antibacterial action versus *E. coli* and *S. aureus*, with IZ diameters of 8.76 ± 1.2 and 9.98 ± 0.6 mm, respectively.	[100]
P(VDF-TrFE)	ZnO	Scaffold	LTE	*S. aureus* and *P. aeruginosa* biofilm formation was inhibited by the ZnO/P(VDF-TrFE) electrospun fiber meshes, and the cell/scaffold structures were effective to hinder *S. aureus* adhesion, and *P. aeruginosa* invasiveness, regardless of the scaffold type.	[101]
CS	TiO_2_	Scaffold	Wound healing	In nursing care, the produced CS/Sr-TiO_2_ nanocomposite coating exhibits increased antibacterial performance as well as superior joint wound healing characteristics.	[102]
CS/PVA	TiO_2_-Ag	Nanofibers	Biomedical	The nanofibers had antibacterial performance versus *S. aureus* and *E. coli* of 99 and 98 percent, respectively.	[103]
GG	TiO_2_	Film	Wound healing	Antibacterial performance of GG+TiO_2_-NTs (20 w/w percent) was measured as a 16 ± 0.06, 16 ± 0.06, 14 ± 0.06, and 12 ± 0.25 mm IZ versus *S. aureus*, *Streptococcus*, *E. coli*, and *P. aeruginosa*, respectively.	[104]
PVA/Plur/PEI	TiO_2_	Nanofibers	Wound healing	The antibacterial effects of the PVA-Plur-PEI/TiO_2_ nanofibers are more effective versus Gram-positive bacteria compared to the PVA-Plur-PEI nanofibers	[105]
PCL	CS-tetracycline HCL	Scaffold	TE	The PCL/CS and nHA/PCL/CS scaffolds were found to be ineffective against *E. coli* and *Bacillus cereus*. Because the CS level is low, blending PCL with it has no antibacterial characteristics. Tetracycline HCL encapsulated in the scaffold improved the blend’s antibacterial characteristics and demonstrated excellent results against both Gram-positive and Gram-negative bacteria.	[106]
SF	CS	Scaffold	TE	When CS was used in higher concentrations in the blends, it had an antimicrobial impact. In addition, as compared to blended scaffolds, CS was more effective at inhibiting *S. aureus* development.	[107]
PCL	CS	Membranes	Biomedical	*S. mutans* and *A. actinomycetemcomitans* bacteria were resistant to CS. The antibacterial properties of CS were affected by the addition of PCL.	[108]
Gel/CS	CS- cinnamaldehyde	Membranes	Wound dressing	The antibacterial behavior of CS/Gel was moderate, with a considerable rise in inhibitory potential as the cinnamaldehyde concentration was elevated.	[109]
PCL	CS-CMC	Scaffold	VTE	Both *S. aureus* and *E. coli* showed no bactericidal effects towards the PCL nanofibrous membrane. A smaller number of bacteria were destroyed by the PCL/CMC nanofibrous membranes. On the other hand, a large number of dead bacteria were found on the PCL/CS surface.	[110]
TiO_2_	CS	Nanocomposites	TE	In the same amount, a neat nano-TiO_2_ impregnated disk exhibits no zone of inhibition; whereas a TiO_2_–CS nanocomposite reveals an inhibition.	[111]
CS	CS-Gentamicin	Film	Biomedical	In comparison with the neat CS film, the CFU of *S. aureus* and *E. coli* on Col-GT’s agar culture dish were substantially lower than CS specimens. Compared to the CS film, the CS-GT film has a markedly improved antimicrobial performance. The CFU on the agar culture dish of CS-GT are much lower than on the agar culture dish of the CS film.	[112]
PEGF	CS	Film	Wound dressing	The antibacterial behavior of the blend films versus *P. aeruginosa* and *S. aureus* was impressive (Kill percent > 99.76 ± 0.16%).	[113]
PU	CS	Film	Medical	*S. aureus* and *P. aeruginosa* bacteria had dramatically improved antimicrobial property after being treated with CS. After CS treatment of PU films, the number of bacterium colonies was reduced to around 10^2^–10^5^ CFU/mL, and the amount of connected live bacteria dropped considerably.	[114]
PCL	CS	Scaffold	Wound dressing	The antibacterial behavior of the PCL-CS scaffolds was remarkable, with obvious IZ values of 13.97 ± 0.12 mm and 12.11 ± 0.13 mm versus *E. coli* and *S. aureus*, respectively, that were comparable to the native CS.	[115]
PEGDA	CS-TCS-Trp-rich peptides	Hydrogels	Wound dressing	The specimen with the appropriate formula of 15% PEGDA and 2% CS or TCS had outstanding mechanical adhesiveness, maintained antibacterial peptide and plasmid DNA release, and dramatically enhanced in vivo wound healing.	[116]

Ag@CMs: Silver-loaded CS microspheres; Al: Aluminum; Alg: Alginate; APA: 6-aminopenicillanic acid; AuNR: Gold nanorods; AZO: Al-doped ZnO; BC: Bacterial cellulose; BTE: Bone tissue engineering; BG: Bioactive glass; CFU: Colony forming units; CMC: Carboxymethyl cellulose; Col: collagen; CS: Chitosan; DBM: Dental barrier membranes; DEG: Diethyleneglycol; ESM: Eggshell membrane; fMWCNTs: Functionalized multi-wall carbon nanotubes; F. vulgaris: Falcaria vulgaris; G. africana: Galenia africana; Gel: Gelatin; GG: Gellan gum; GO: Graphene oxide; GO-COOH: Carboxylated graphene oxide; HA: Hyaluronic acid; HAp: Hydroxyapatite; H. hemerocallidea: Hypoxis hemerocallidea; LTE: Lung tissue engineering; MBGs: Mesoporous bioactive glasses; MDR: Remedying multidrug-resistant; Mg: Magnesium; MRSA: Methicillin-resistant Staphylococcus aureus; NP: Nanoparticles; P. aeruginosa: Pseudomonas aeruginosa; PAH: Poly allylamine hydrochloride; PANI: Polyaniline; PCL: Polycaprolactone; PEG: Polyethylene glycol; PEGDA: Poly (ethylene glycol) diacrylate; PEGF: Polyethylene glycol fumarate; PEI: Polyethyleneimine; PEO: Polyethylene oxide; PGF: Phosphate-based glass fibers; PHBV: Poly-(3-hydroxybutyrate-co-3- hydroxyvalerate); PLA: Poly (lactide acid); Plur: Pluronic F127; PU: Polyurethane; PVA: Poly (vinyl alcohol); P(VDF-TrFE): Poly (vinylidene fluoride-co-trifluoroethylene); SA: Sodium alginate; SF: Silk/fibroin; SSA: Specific surface area; STE: Soft tissue engineering; Sr: Strontium; Step: Streptococcus; TCS: Thiolated chitosan; THR: Total hip replacement; TiO_2_: Titanium dioxide; TKA: Total knee arthroplasty; VTE: vascular tissue engineering; WS: wollastonite; ZnO: Zinc oxide.

**Table 2:** Printing inks containing antibacterial agents.

Material	Antibacterial Agent	3D Printing Method	Antibacterial Assay	Cellular Assay and Cell Type	App	Ref.
PCL	Silver, using 1% and 3% silver nitrate	FDM based	Scaffolds encapsulated with 3 wt. % Ag presented large IZ, while no clear IZ detected for PCL and 1wt. % Ag	Higher cell response for 1 wt. % Ag than PCL, while 3 wt. % Ag presented poor cell viability.Cell type: hFOB	BTE	[37]
PCL-PPSu	Ag	Extrusion-based	Copolymers encapsulated with AgNO_3_ presented antimicrobial performance toward *E. coli, P. aeruginosa, S. aureus,* and *C. albicans*	Encapsulation of a high amount of AgNO_3_ led to reduction in viability, owing to the release of a high amount of Ag^+^ ions from the scaffold to the surrounding environment. Cell type: HDF	STE	[36]
CS/PEO/GP	ZnO	BioX bioprinter	ZnONPs with a size of 90 nm treated with UV presented the greatest antibacterial performance	-	TE	[130]
PCL	CS	Extrusion-based	Lower bacteria growth rate was detected for CS-treated scaffolds, where the Mw of chitosan has a less significant effect on antibacterial performance	CS-treated scaffolds exhibited excellent cell attachment and cell viability. Cell type: L929 fibroblasts	TE	[39]
PLA	Ponericin	FDM	Both Gram-positive and negative bacteria were significantly inhibited up to 24 h and the IZ remained stable up to 72 h	The scaffolds presented excellent MC3T3-E1 cell attachment, spread, and growth. Cell type: MC3T3-E1	BTE	[40]
3DPZS	Ag	Extrusion-based	Ag-3DPZS presented excellent antibacterial behavior, owing to the generation of Ag into the surrounding environment	No significant difference between the Ag-3DPZS sample and the control sample was observed, implying the non-cytotoxicity of Ag encapsulated with a zeolite scaffold. Cell type: MC3T3-E1	BTE	[131]
β-TCP	Ag	Printing machine with a sprayer	The scaffolds encapsulated with Ag@GO exhibited excellent antibacterial performance toward *E. coli*	The scaffolds encapsulated with Ag@GO escalated ALP and osteogenic differentiation Cell type: rBMSCs	BTE	[132]
PCL- PDA	Ag	FDM	PCL/PDA/AgNPs scaffolds could reduce bacterial attachment and regeneration, while increasing the diameter of the IZ	PCL/PDA/AgNPs scaffolds presented a suitable cell response. Cell type: BMSCs	BTE	[133]
PAM/ HPMC and CS	Ag	FDM	No IZs around the HPMC/CS-encapsulated hydrogel dressings were found, while the AgNP-crosslinked dressings presented obvious IZs toward *S. aureus* and *E. coli*	All hydrogel dressings presented good L929 cell viability, and the release of Ag from the crosslinked dressing did not induce cytotoxicity. Cell type: L929	Wound dressing	[134]
iPDMS and silicone oil	Ag	Bioprinter	iPDMS/AgNPs could significantly prevent wound dressing infection	Excellent biocompatibility, promoting neo-epithelial and granulation tissue formation to accelerate wound healing in vivo. Cell type: Fibroblast	Wound dressing	[135]
ZrO_2_	ZnO	3D printer (Makerbot Z18, America)	The ZrO_2_-ZnO ceramics had a substantial antibacterial performance	ZrO_2_-ZnO ceramics presented high cell viability (around 80%). Cell type: MC3T3-E1	Hip joint	[136]
PLGA	ZIF-8, Copper	Extrusion-based	PLGA/Cu(I)@ZIF-8 scaffolds destroyed *S. aureus* bacteria, and bacteria numbers were considerably diminished in infected rats after implantation with the scaffolds	The cells were well spread and attached with a high growth rate on PLGA/Cu(I)@ZIF-8 scaffolds. Cell type: mMSC	BTE	[137]
PCL/ Lidocaine	Ag	Extrusion-based	Scaffolds loaded with Ag presented excellent IZs towards *S. aureus* and *E. coli* in a dose-dependent manner	Ag-encapsulated scaffolds showed a toxic effect to MC3T3 cells, as a result of dual-released lidocaine and Ag, while no cytotoxicity effect was detected for the neat lidocaine- or Ag_3_PO_4_-loaded scaffolds. Cell type: HFFs and MC3T3	Infection prevention and pain relief	[138]

Ag: Silver; Ag@GO: Silver/graphene oxide nanocomposite; App: Application; BMSCs: Bone marrow mesenchymal stem cells; BTE: Bone tissue engineering; CS: Chitosan; IZ: Inhibition zone; FDM: Fused deposition modeling; GO: Graphene oxide; GP: Glycerol phosphate; HDF: Human dermal fibroblast; HFFs: Human foreskin fibroblasts; hFOB: Human fetal osteoblast; iPDMS: Poly dimethylsiloxane; MGO: Magnesium oxide; PCL: Polycaprolactone; PCL-PPSu: polycaprolactone-block-poly(1,3-propylene succinate); PDA: Polydopamine; PEO: Polyethylene oxide; PLA: Polylactic acid; PLGA: Poly (lactide-*co*-glycolide); rBMSCs: Rabbit bone marrow stromal cells; STE: Skin tissue engineering; TE: Tissue engineering; 3DPZS: Zincosilicate zeolite scaffolds; ZIF-8: Zeolitic imidazolate frameworks.

## References

[1] Chen X.B. (2019). Extrusion Bioprinting of Scaffolds for Tissue Engineering Applications.

[2] Sadeghianmaryan A., Naghieh S., Yazdanpanah Z., Sardroud H.A., Sharma N., Wilson L.D., Chen X. (2022). Fabrication of chitosan/alginate/hydroxyapatite hybrid scaffolds using 3D printing and impregnating techniques for potential cartilage regeneration. Int. J. Biol. Macromol..

[3] Zimmerling A., Zhou Y., Chen X. (2021). Bioprinted constructs for respiratory tissue engineering. Bioprinting.

[4] Delkash Y., Gouin M., Rimbeault T., Mohabatpour F., Papagerakis P., Maw S., Chen X. (2021). Bioprinting and In Vitro Characterization of an Eggwhite-Based Cell-Laden Patch for Endothelialized Tissue Engineering Applications. J. Funct. Biomater..

[5] Soleymani Eil Bakhtiari S., Bakhsheshi-Rad H.R., Karbasi S., Razzaghi M., Tavakoli M., Ismail A.F., Sharif S., Rama Krishna S., Chen X., Berto F. (2021). 3-Dimensional Printing of Hydrogel-Based Nanocomposites: A Comprehensive Review on the Technology Description, Properties, and Applications. Adv. Eng. Mater..

[6] Fu Z., Naghieh S., Xu C., Wang C., Sun W., Chen D.X. (2021). Printability in extrusion bioprinting. Biofabrication.

[7] Sadeghianmaryan A., Naghieh S., Sardroud H.A., Yazdanpanah Z., Soltani Y.A., Sernaglia J., Chen X. (2020). Extrusion-based printing of chitosan scaffolds and their in vitro characterization for cartilage tissue engineering. Int. J. Biol. Macromol..

[8] You F., Wu X., Kelly M., Chen X. (2020). Bioprinting and in vitro characterization of alginate dialdehyde–gelatin hydrogel bio-ink. Bio-Des. Manuf..

[9] Soleymani Eil Bakhtiari S., Bakhsheshi-Rad H.R., Karbasi S., Tavakoli M., Razzaghi M., Ismail A.F., RamaKrishna S., Berto F. (2020). Polymethyl Methacrylate-Based Bone Cements Containing Carbon Nanotubes and Graphene Oxide: An Overview of Physical, Mechanical, and Biological Properties. Polymers.

[10] You F., Chen D.X., Cooper D.M.L., Chang T., Eames F.B. (2018). Homogeneous hydroxyapatite/alginate composite hydrogel promotes calcified cartilage matrix deposition with potential for three-dimensional bioprinting. Biofabrication.

[11] Ning L., Sun H., Lelong T., Guilloteau R., Zhu N., Schreyer D.J., Chen X. (2018). 3D bioprinting of scaffolds with living Schwann cells for potential nerve tissue engineering applications. Biofabrication.

[12] Bakhsheshi-Rad H.R., Ismail A.F., Aziz M., Akbari M., Hadisi Z., Omidi M., Chen X. (2020). Development of the PVA/CS nanofibers containing silk protein sericin as a wound dressing: In vitro and in vivo assessment. Int. J. Biol. Macromol..

[13] Bakhsheshi-Rad H., Hadisi Z., Ismail A., Aziz M., Akbari M., Berto F., Chen X. (2020). In vitro and in vivo evaluation of chitosan-alginate/gentamicin wound dressing nanofibrous with high antibacterial performance. Polym. Test..

[14] Jammalamadaka U., Tappa K. (2018). Recent Advances in Biomaterials for 3D Printing and Tissue Engineering. J. Funct. Biomater..

[15] Klebe R.J. (1988). Cytoscribing: A method for micropositioning cells and the construction of two- and three-dimensional synthetic tissues. Exp. Cell Res..

[16] Gu Z., Fu J., Lin H., He Y. (2020). Development of 3D bioprinting: From printing methods to biomedical applications. Asian J. Pharm. Sci..

[17] Muhammad M.H., Idris A.L., Fan X., Guo Y., Yu Y., Jin X., Qiu J., Guan X., Huang T. (2020). Beyond Risk: Bacterial Biofilms and Their Regulating Approaches. Front. Microbiol..

[18] Singh A., Dubey A.K. (2018). Various Biomaterials and Techniques for Improving Antibacterial Response. ACS Appl. Biol. Mater..

[19] Suresh A.K., Pelletier D.A., Wang W., Moon J.-W., Gu B., Mortensen N.P., Allison D.P., Joy D.C., Phelps T.J., Doktycz M.J. (2010). Silver Nanocrystallites: Biofabrication using *Shewanella oneidensis,* and an Evaluation of Their Comparative Toxicity on Gram-negative and Gram-positive Bacteria. Environ. Sci. Technol..

[20] Spieser H., Jardin A., Deganello D., Gethin D., Bras J., Denneulin A. (2021). Rheology of cellulose nanofibrils and silver nanowires for the development of screen-printed antibacterial surfaces. J. Mater. Sci..

[21] Hasan K.M., Pervez M., Talukder M., Sultana M., Mahmud S., Meraz M., Bansal V., Genyang C. (2019). A novel coloration of polyester fabric through green silver nanoparticles (G-AgNPs@ PET). Nanomaterials.

[22] Setayeshmehr M., Hafeez S., van Blitterswijk C., Moroni L., Mota C., Baker M. (2021). Bioprinting Via a Dual-Gel Bioink Based on Poly(Vinyl Alcohol) and Solubilized Extracellular Matrix towards Cartilage Engineering. Int. J. Mol. Sci..

[23] Valot L., Martinez J., Mehdi A., Subra G. (2019). Chemical insights into bioinks for 3D printing. Chem. Soc. Rev..

[24] Rastin H., Ramezanpour M., Hassan K., Mazinani A., Tung T.T., Vreugde S., Losic D. (2021). 3D bioprinting of a cell-laden antibacterial polysaccharide hydrogel composite. Carbohydr. Polym..

[25] Guzmán-Soto I., McTiernan C., Gonzalez-Gomez M., Ross A., Gupta K., Suuronen E.J., Mah T.-F., Griffith M., Alarcon E.I. (2021). Mimicking biofilm formation and development: Recent progress in in vitro and in vivo biofilm models. iScience.

[26] Jacobs A., Renaudin G., Forestier C., Nedelec J.-M., Descamps S. (2020). Biological properties of copper-doped biomaterials for orthopedic applications: A review of antibacterial, angiogenic and osteogenic aspects. Acta Biomater..

[27] Dubinenko G., Zinoviev A., Bolbasov E., Kozelskaya A., Shesterikov E., Novikov V., Tverdokhlebov S. (2021). Highly filled poly (l-lactic acid)/hydroxyapatite composite for 3D printing of personalized bone tissue engineering scaffolds. J. Appl. Polym. Sci..

[28] Messaoudi O., Henrionnet C., Bourge K., Loeuille D., Gillet P., Pinzano A. (2020). Stem Cells and Extrusion 3D Printing for Hyaline Cartilage Engineering. Cells.

[29] Zhang J., Yun S., Karami A., Jing B., Zannettino A., Du Y., Zhang H. (2020). 3D printing of a thermosensitive hydrogel for skin tissue engineering: A proof of concept study. Bioprinting.

[30] Mondal S., Nguyen T.P., Pham V.H., Hoang G., Manivasagan P., Kim M.H., Nam S.Y., Oh J. (2020). Hydroxyapatite nano bioceramics optimized 3D printed poly lactic acid scaffold for bone tissue engineering application. Ceram. Int..

[31] Babilotte J., Martin B., Guduric V., Bareille R., Agniel R., Roques S., Héroguez V., Dussauze M., Gaudon M., Le Nihouannen D. (2021). Development and characterization of a PLGA-HA composite material to fabricate 3D-printed scaffolds for bone tissue engineering. Mater. Sci. Eng. C.

[32] Zhao X., Li P., Guo B., Ma P.X. (2015). Antibacterial and conductive injectable hydrogels based on quaternized chitosan-graft-polyaniline/oxidized dextran for tissue engineering. Acta Biomater..

[33] Cai J., Liu R. (2020). Introduction to Antibacterial Biomaterials. Biomater. Sci..

[34] Lu H., Liu Y., Guo J., Wu H., Wang J., Wu G. (2016). Biomaterials with Antibacterial and Osteoinductive Properties to Repair Infected Bone Defects. Int. J. Mol. Sci..

[35] Hasan J., Crawford R., Ivanova E.P. (2013). Antibacterial surfaces: The quest for a new generation of biomaterials. Trends Biotechnol..

[36] Afghah F., Ullah M., Zanjani J.S.M., Süt P.A., Sen O., Emanet M., Okan B.S., Culha M., Menceloglu Y., Yildiz M. (2020). 3D printing of silver-doped polycaprolactone-poly propylene succinate composite scaffolds for skin tissue engineering. Biomed. Mater..

[37] Radhakrishnan S., Nagarajan S., Belaid H., Farha C., Iatsunskyi I., Coy E., Soussan L., Huon V., Bares J., Belkacemi K. (2021). Fabrication of 3D printed antimicrobial polycaprolactone scaffolds for tissue engineering applications. Mater. Sci. Eng. C.

[38] Zhu T., Zhu M., Zhu Y. (2020). Fabrication of forsterite scaffolds with photothermal-induced antibacterial activity by 3D printing and polymer-derived ceramics strategy. Ceram. Int..

[39] Tardajos M.G., Cama G., Dash M., Misseeuw L., Gheysens T., Gorzelanny C., Coenye T., Dubruel P. (2018). Chitosan functionalized poly-ε-caprolactone electrospun fibers and 3D printed scaffolds as antibacterial materials for tissue engineering applications. Carbohydr. Polym..

[40] Kumar R., Umar A., Kumar G., Nalwa H.S. (2017). Antimicrobial properties of ZnO nanomaterials: A review. Ceram. Int..

[41] Bazaka K., Jacob M., Crawford R.J., Ivanova E.P. (2012). Efficient surface modification of biomaterial to prevent biofilm formation and the attachment of microorganisms. Appl. Microbiol. Biotechnol..

[42] Arciola C.R., Campoccia D., Montanaro L. (2018). Implant infections: Adhesion, biofilm formation and immune evasion. Nat. Rev. Microbiol..

[43] Bakhsheshi-Rad H.R., Hamzah E., Ismail A.F., Aziz M., Kasiri-Asgarani M., Ghayour H., Razzaghi M., Hadisi Z. (2017). In vitro corrosion behavior, bioactivity, and antibacterial performance of the silver-doped zinc oxide coating on magnesium alloy. Mater. Corros..

[44] Campoccia D., Montanaro L., Arciola C.R. (2013). A review of the biomaterials technologies for infection-resistant surfaces. Biomaterials.

[45] Lv W., Luo J., Deng Y., Sun Y. (2013). Biomaterials immobilized with chitosan for rechargeable antimicrobial drug delivery. J. Biomed. Mater. Res. Part A.

[46] Choudhury H., Pandey M., Lim Y.Q., Low C.Y., Lee C.T., Marilyn T.C.L., Loh H.S., Lim Y.P., Bhattamishra S.K., Kesharwani P. (2020). Silver nanoparticles: Advanced and promising technology in diabetic wound therapy. Mater. Sci. Eng. C.

[47] Cui Y., Zhao Y., Tian Y., Zhang W., Lü X., Jiang X. (2012). The molecular mechanism of action of bactericidal gold nanoparticles on Escherichia coli. Biomaterials.

[48] Mitik-Dineva N., Wang J., Truong V.K., Stoddart P., Malherbe F., Crawford R., Ivanova E.P. (2009). Escherichia coli, Pseudomonas aeruginosa, and Staphylococcus aureus Attachment Patterns on Glass Surfaces with Nanoscale Roughness. Curr. Microbiol..

[49] Sahithi K., Swetha M., Prabaharan M., Moorthi A., Saranya N., Ramasamy K., Srinivasan N., Partridge N., Selvamurugan N. (2010). Synthesis and Characterization of NanoscaleHydroxyapatite-Copper for Antimicrobial Activity Towards Bone Tissue Engineering Applications. J. Biomed. Nanotechnol..

[50] Mishra S.K., Mary D.S., Kannan S. (2017). Copper incorporated microporous chitosan-polyethylene glycol hydrogels loaded with naproxen for effective drug release and anti-infection wound dressing. Int. J. Biol. Macromol..

[51] Baino F. (2020). Copper-Doped Ordered Mesoporous Bioactive Glass: A Promising Multifunctional Platform for Bone Tissue Engineering. Bioengineering.

[52] Azeena S., Subhapradha N., Selvamurugan N., Narayan S., Srinivasan N., Murugesan R., Chung T., Moorthi A. (2017). Antibacterial activity of agricultural waste derived wollastonite doped with copper for bone tissue engineering. Mater. Sci. Eng. C.

[53] Li X., Wang Y., Guo M., Wang Z., Shao N., Zhang P., Chen X., Huang Y. (2018). Degradable Three Dimensional-Printed Polylactic Acid Scaffold with Long-Term Antibacterial Activity. ACS Sustain. Chem. Eng..

[54] Wu C., Zhou Y., Xu M., Han P., Chen L., Chang J., Xiao Y. (2013). Copper-containing mesoporous bioactive glass scaffolds with multifunctional properties of angiogenesis capacity, osteostimulation and antibacterial activity. Biomaterials.

[55] Abou Neel E.A., Ahmed I., Pratten J., Nazhat S.N., Knowles J.C. (2005). Characterisation of antibacterial copper releasing degradable phosphate glass fibres. Biomaterials.

[56] Li J., Zhai D., Lv F., Yu Q., Ma H., Yin J., Yi Z., Liu M., Chang J., Wu C. (2016). Preparation of copper-containing bioactive glass/eggshell membrane nanocomposites for improving angiogenesis, antibacterial activity and wound healing. Acta Biomater..

[57] Yang Y., Zheng K., Liang R., Mainka A., Taccardi N., Roether J.A., Detsch R., Goldmann W.H., Virtanen S., Boccaccini A.R. (2017). Cu-releasing bioactive glass/polycaprolactone coating on Mg with antibacterial and anticorrosive properties for bone tissue engineering. Biomed. Mater..

[58] Xu S., Wu Q., Guo Y., Ning C., Dai K. (2021). Copper containing silicocarnotite bioceramic with improved mechanical strength and antibacterial activity. Mater. Sci. Eng. C.

[59] Menazea A., Ahmed M. (2020). Synthesis and antibacterial activity of graphene oxide decorated by silver and copper oxide nanoparticles. J. Mol. Struct..

[60] Tripathi A., Saravanan S., Pattnaik S., Moorthi A., Partridge N., Selvamurugan N. (2012). Bio-composite scaffolds containing chitosan/nano-hydroxyapatite/nano-copper–zinc for bone tissue engineering. Int. J. Biol. Macromol..

[61] Augustine R., Kalarikkal N., Thomas S. (2016). Electrospun PCL membranes incorporated with biosynthesized silver nanoparticles as antibacterial wound dressings. Appl. Nanosci..

[62] Saini R.K., Bagri L.P., Bajpai A.K. (2019). Nano-silver hydroxyapatite based antibacterial 3D scaffolds of gelatin/alginate/poly (vinyl alcohol) for bone tissue engineering applications. Colloids Surf. B Biointerfaces.

[63] Wang X., Cheng F., Gao J., Wang L. (2015). Antibacterial wound dressing from chitosan/polyethylene oxide nanofibers mats embedded with silver nanoparticles. J. Biomater. Appl..

[64] Shameli K., Ahmad M.B., Yunus W.M., Ibrahim N.A., Rahman R.A., Jokar M., Darroudi M. (2010). Silver/poly (lactic acid) nanocomposites: Preparation, characterization, and antibacterial activity. Int. J. Nanomed..

[65] Hosseini H., Zirakjou A., Goodarzi V., Mousavi S.M., Khonakdar H.A., Zamanlui S. (2020). Lightweight aerogels based on bacterial cellulose/silver nanoparticles/polyaniline with tuning morphology of polyaniline and application in soft tissue engineering. Int. J. Biol. Macromol..

[66] Vaidhyanathan B., Vincent P., Vadivel S., Karuppiah P., Al-Dhabi N.A., Sadhasivam D.R., Vimalraj S., Saravanan S. (2021). Fabrication and Investigation of the Suitability of Chitosan-Silver Composite Scaffolds for Bone Tissue Engineering Applications. Process Biochem..

[67] Yahyaei B., Peyvandi N., Akbari H., Arabzadeh S., Afsharnezhad S., Ajoudanifar H., Pourali P. (2016). Production, assessment, and impregnation of hyaluronic acid with silver nanoparticles that were produced by Streptococcus pyogenes for tissue engineering applications. Appl. Biol. Chem..

[68] Patil S., Singh N. (2019). Antibacterial silk fibroin scaffolds with green synthesized silver nanoparticles for osteoblast proliferation and human mesenchymal stem cell differentiation. Colloids Surf. B Biointerfaces.

[69] Wadke P., Chhabra R., Jain R., Dandekar P. (2017). Silver-embedded starch-based nanofibrous mats for soft tissue engineering. Surf. Interfaces.

[70] Stanić V., Janaćković D., Dimitrijević S., Tanasković S.B., Mitrić M., Pavlović M.S., Krstić A., Jovanović D., Raičević S. (2011). Synthesis of antimicrobial monophase silver-doped hydroxyapatite nanopowders for bone tissue engineering. Appl. Surf. Sci..

[71] Bakhsheshi-Rad H.R., Dayaghi E., Ismail A.F., Aziz M., Akhavan-Farid A., Chen X. (2019). Synthesis and in-vitro characterization of biodegradable porous magnesium-based scaffolds containing silver for bone tissue engineering. Trans. Nonferrous Met. Soc. China.

[72] Bakhsheshi-Rad H.R., Ismail A.F., Aziz M., Akbari M., Hadisi Z., Khoshnava S.M., Pagan E., Chen X. (2020). Co-incorporation of graphene oxide/silver nanoparticle into poly-L-lactic acid fibrous: A route toward the development of cytocompatible and antibacterial coating layer on magnesium implants. Mater. Sci. Eng. C.

[73] Niu X., Wei Y., Liu Q., Yang B., Ma N., Li Z., Zhao L., Chen W., Huang D. (2020). Silver-loaded microspheres reinforced chitosan scaffolds for skin tissue engineering. Eur. Polym. J..

[74] Marsich E., Bellomo F., Turco G., Travan A., Donati I., Paoletti S. (2013). Nano-composite scaffolds for bone tissue engineering containing silver nanoparticles: Preparation, characterization and biological properties. J. Mater. Sci. Mater. Med..

[75] Saravanan S., Nethala S., Pattnaik S., Tripathi A., Moorthi A., Selvamurugan N. (2011). Preparation, characterization and antimicrobial activity of a bio-composite scaffold containing chitosan/nano-hydroxyapatite/nano-silver for bone tissue engineering. Int. J. Biol. Macromol..

[76] Xing Z.-C., Chae W.-P., Baek J.-Y., Choi M.-J., Jung Y., Kang I.-K. (2010). In Vitro Assessment of Antibacterial Activity and Cytocompatibility of Silver-Containing PHBV Nanofibrous Scaffolds for Tissue Engineering. Biomacromolecules.

[77] Lim M.M., Sultana N. (2016). In vitro cytotoxicity and antibacterial activity of silver-coated electrospun polycaprolactone/gelatine nanofibrous scaffolds. 3 Biotech.

[78] Sumitha M.S., Shalumon K.T., Sreeja V.N., Jayakumar R., Nair S.V., Menon D. (2012). Biocompatible and Antibacterial Nanofibrous Poly(ϵ-caprolactone)-Nanosilver Composite Scaffolds for Tissue Engineering Applications. J. Macromol. Sci. Part A.

[79] Ribeiro M., Ferraz M.P., Monteiro F., Fernandes M.H., Beppu M.M., Mantione D., Sardon H. (2017). Antibacterial silk fibroin/nanohydroxyapatite hydrogels with silver and gold nanoparticles for bone regeneration. Nanomed. Nanotechnol. Biol. Med..

[80] Chitra G., Franklin D., Sudarsan S., Sakthivel M., Guhanathan S. (2018). Noncytotoxic silver and gold nanocomposite hydrogels with enhanced antibacterial and wound healing applications. Polym. Eng. Sci..

[81] Li Q., Lu F., Zhou G., Yu K., Lu B., Xiao Y., Dai F., Wu D., Lan G. (2017). Silver Inlaid with Gold Nanoparticle/Chitosan Wound Dressing Enhances Antibacterial Activity and Porosity, and Promotes Wound Healing. Biomacromolecules.

[82] Prakash J., Prema D., Venkataprasanna K., Balagangadharan K., Selvamurugan N., Venkatasubbu G.D. (2020). Nanocomposite chitosan film containing graphene oxide/hydroxyapatite/gold for bone tissue engineering. Int. J. Biol. Macromol..

[83] Russo T., Gloria A., De Santis R., D’Amora U., Balato G., Vollaro A., Oliviero O., Improta G., Triassi M., Ambrosio L. (2017). Preliminary focus on the mechanical and antibacterial activity of a PMMA-based bone cement loaded with gold nanoparticles. Bioact. Mater..

[84] Menazea A., Ahmed M. (2020). Wound healing activity of Chitosan/Polyvinyl Alcohol embedded by gold nanoparticles prepared by nanosecond laser ablation. J. Mol. Struct..

[85] Yang X., Yang J., Wang L., Ran B., Jia Y., Zhang L., Yang G., Shao H., Jiang X. (2017). Pharmaceutical Intermediate-Modified Gold Nanoparticles: Against Multidrug-Resistant Bacteria and Wound-Healing Application via an Electrospun Scaffold. ACS Nano.

[86] Haidari H., Kopecki Z., Bright R., Cowin A.J., Garg S., Goswami N., Vasilev K. (2020). Ultrasmall AgNP-impregnated biocompatible hydrogel with highly effective biofilm elimination properties. ACS Appl. Mater. Interfaces.

[87] Zangeneh M.M., Saneei S., Zangeneh A., Toushmalani R., Haddadi A., Almasi M., Amiri-Paryan A. (2019). Preparation, characterization, and evaluation of cytotoxicity, antioxidant, cutaneous wound healing, antibacterial, and antifungal effects of gold nanoparticles using the aqueous extract of Falcaria vulgaris leaves. Appl. Organomet. Chem..

[88] Elbagory A.M., Meyer M., Cupido C.N., Hussein A.A. (2017). Inhibition of Bacteria Associated with Wound Infection by Biocompatible Green Synthesized Gold Nanoparticles from South African Plant Extracts. Nanomaterials.

[89] Mahmoud N.N., Hikmat S., Abu Ghith D., Hajeer M., Hamadneh L., Qattan D., Khalil E.A. (2019). Gold nanoparticles loaded into polymeric hydrogel for wound healing in rats: Effect of nanoparticles’ shape and surface modification. Int. J. Pharm..

[90] Varaprasad K., Raghavendra G.M., Jayaramudu T., Seo J. (2015). Nano zinc oxide–sodium alginate antibacterial cellulose fibres. Carbohydr. Polym..

[91] Balaure P.C., Holban A.M., Grumezescu A.M., Mogoşanu G.D., Bălşeanu T.A., Stan M.S., Dinischiotu A., Volceanov A., Mogoantă L. (2019). In vitro and in vivo studies of novel fabricated bioactive dressings based on collagen and zinc oxide 3D scaffolds. Int. J. Pharm..

[92] Rakhshaei R., Namazi H., Hamishehkar H., Kafil H.S., Salehi R. (2019). *In situ* synthesized chitosan–gelatin/ZnO nanocomposite scaffold with drug delivery properties: Higher antibacterial and lower cytotoxicity effects. J. Appl. Polym. Sci..

[93] Trandafilović L.V., Božanić D.K., Dimitrijević-Branković S., Luyt A.S., Djoković V. (2012). Fabrication and antibacterial properties of ZnO–alginate nanocomposites. Carbohydr. Polym..

[94] Chen J., Zhang X., Cai H., Chen Z., Wang T., Jia L., Wang J., Wan Q., Pei X. (2016). Osteogenic activity and antibacterial effect of zinc oxide/carboxylated graphene oxide nanocomposites: Preparation and in vitro evaluation. Colloids Surf. B Biointerfaces.

[95] Shalumon K.T., Anulekha K.H., Nair S.V., Nair S.V., Chennazhi K.P., Jayakumar R. (2011). Sodium alginate/poly (vinyl alcohol)/nano ZnO composite nanofibers for antibacterial wound dressings. Int. J. Biol. Macromol..

[96] Farhoudian S., Yadollahi M., Namazi H. (2016). Facile synthesis of antibacterial chitosan/CuO bio-nanocomposite hydrogel beads. Int. J. Biol. Macromol..

[97] Shrestha B.K., Shrestha S., Tiwari A.P., Kim J.-I., Ko S.W., Kim H.-J., Park C.H., Kim C.S. (2017). Bio-inspired hybrid scaffold of zinc oxide-functionalized multi-wall carbon nanotubes reinforced polyurethane nanofibers for bone tissue engineering. Mater. Des..

[98] Saxena V., Pandey L.M. (2019). Synthesis, characterization and antibacterial activity of aluminum doped zinc oxide. Mater. Today Proc..

[99] Felice B., Sánchez M.A., Socci M.C., Sappia L.D., Gómez M.I., Cruz M.K., Felice C.J., Martí M., Pividori M.I., Simonelli G. (2018). Controlled degradability of PCL-ZnO nanofibrous scaffolds for bone tissue engineering and their antibacterial activity. Mater. Sci. Eng. C.

[100] Augustine R., Malik H., Singhal D.K., Mukherjee A., Malakar D., Kalarikkal N., Thomas S. (2014). Electrospun polycaprolactone/ZnO nanocomposite membranes as biomaterials with antibacterial and cell adhesion properties. J. Polym. Res..

[101] Azimi B., Sorayani Bafqi M.S., Fusco A., Ricci C., Gallone G., Bagherzadeh R., Donnarumma G., Uddin M.J., Latifi M., Lazzeri A. (2020). Electrospun ZnO/poly (vinylidene fluoride-trifluoroethylene) scaffolds for lung tissue engineering. Tissue Eng. Part A.

[102] Chen L., Pan H., Zhuang C., Peng M., Zhang L. (2020). Joint wound healing using polymeric dressing of chitosan/strontium-doped titanium dioxide with high antibacterial activity. Mater. Lett..

[103] Son B., Yeom B.-Y., Song S.H., Lee C.-S., Hwang T.S. (2009). Antibacterial electrospun chitosan/poly(vinyl alcohol) nanofibers containing silver nitrate and titanium dioxide. J. Appl. Polym. Sci..

[104] Razali M.H., Ismail N.A., Amin K.A.M. (2020). Titanium dioxide nanotubes incorporated gellan gum bio-nanocomposite film for wound healing: Effect of TiO2 nanotubes concentration. Int. J. Biol. Macromol..

[105] El-Aassar M.R., El Fawal G.F., El-Deeb N.M., Hassan H.S., Mo X., El-Aassar M. (2016). Electrospun Polyvinyl Alcohol/ Pluronic F127 Blended Nanofibers Containing Titanium Dioxide for Antibacterial Wound Dressing. Appl. Biochem. Biotechnol..

[106] Mad Jin R., Sultana N., Baba S., Hamdan S., Ismail A.F. (2015). Porous PCL/chitosan and nHA/PCL/ chitosan scaffolds for tissue engineering applications: Fabrication and evaluation. J. Nanomater..

[107] Bhardwaj N., Kundu S.C. (2011). Silk fibroin protein and chitosan polyelectrolyte complex porous scaffolds for tissue engineering applications. Carbohydr. Polym..

[108] Sarasam A.R., Krishnaswamy A.R.K., Madihally S.V. (2006). Blending Chitosan with Polycaprolactone: Effects on Physicochemical and Antibacterial Properties. Biomacromolecules.

[109] Kenawy E., Omer A.M., Tamer T.M., Elmeligy M.A., Eldin M.S.M. (2019). Fabrication of biodegradable gelatin/chitosan/cinnamaldehyde crosslinked membranes for antibacterial wound dressing applications. Int. J. Biol. Macromol..

[110] Wang Y., He C., Feng Y., Yang Y., Wei Z., Zhao W., Zhao C. (2020). A chitosan modified asymmetric small-diameter vascular graft with anti-thrombotic and anti-bacterial functions for vascular tissue engineering. J. Mater. Chem. B.

[111] Kavitha K., Sutha S., Prabhu M., Rajendran V., Jayakumar T. (2013). In situ synthesized novel biocompatible titania–chitosan nanocomposites with high surface area and antibacterial activity. Carbohydr. Polym..

[112] Liu Y., Ji P., Lv H., Qin Y., Deng L. (2017). Gentamicin modified chitosan film with improved antibacterial property and cell biocompatibility. Int. J. Biol. Macromol..

[113] Doulabi A.H., Mirzadeh H., Imani M., Samadi N. (2013). Chitosan/polyethylene glycol fumarate blend film: Physical and antibacterial properties. Carbohydr. Polym..

[114] Kara F., Aksoy E.A., Yuksekdag Z., Hasirci N., Aksoy S. (2014). Synthesis and surface modification of polyurethanes with chitosan for antibacterial properties. Carbohydr. Polym..

[115] Ozkan O., Sasmazel H.T. (2018). Antibacterial Performance of PCL-Chitosan Core–Shell Scaffolds. J. Nanosci. Nanotechnol..

[116] Huang L., Zhu Z., Wu D., Gan W., Zhu S., Li W., Tian J., Li L., Zhou C., Lu L. (2019). Antibacterial poly (ethylene glycol) diacrylate/chitosan hydrogels enhance mechanical adhesiveness and promote skin regeneration. Carbohydr. Polym..

[117] Chatterjee A.K., Chakraborty R., Basu T. (2014). Mechanism of antibacterial activity of copper nanoparticles. Nanotechnology.

[118] Raffi M., Mehrwan S., Bhatti T.M., Akhter J.I., Hameed A., Yawar W., ul Hasan M.M. (2010). Investigations into the antibacterial behavior of copper nanoparticles against Escherichia coli. Ann. Microbiol..

[119] Menazea A., Ahmed M. (2020). Nanosecond laser ablation assisted the enhancement of antibacterial activity of copper oxide nano particles embedded though Polyethylene Oxide/Polyvinyl pyrrolidone blend matrix. Radiat. Phys. Chem..

[120] Lansdown A. (2002). Silver I: Its antibacterial properties and mechanism of action. J. Wound Care.

[121] Patil M., Kim G.-D. (2017). Eco-friendly approach for nanoparticles synthesis and mechanism behind antibacterial activity of silver and anticancer activity of gold nanoparticles. Appl. Microbiol. Biotechnol..

[122] Choi O., Deng K.K., Kim N.J., Ross L., Surampalli R.Y., Hu Z. (2008). The inhibitory effects of silver nanoparticles, silver ions, and silver chloride colloids on microbial growth. Water Res..

[123] Ortiz-Benítez E.A., Velázquez-Guadarrama N., Figueroa N.V.D., Quezada H., Olivares-Trejo J.D.J. (2019). Antibacterial mechanism of gold nanoparticles on *Streptococcus pneumoniae*. Metallomics.

[124] Gu X., Xu Z., Gu L., Xu H., Han F., Chen B., Pan X. (2020). Preparation and antibacterial properties of gold nanoparticles: A review. Environ. Chem. Lett..

[125] Sirelkhatim A., Mahmud S., Seeni A., Kaus N.H.M., Ann L.C., Bakhori S.K.M., Hasan H., Mohamad D. (2015). Review on Zinc Oxide Nanoparticles: Antibacterial Activity and Toxicity Mechanism. Nano-Micro Lett..

[126] Li M., Zhu L., Lin D. (2011). Toxicity of ZnO Nanoparticles to *Escherichia coli*: Mechanism and the Influence of Medium Components. Environ. Sci. Technol..

[127] Prakash J., Cho J., Mishra Y.K. (2021). Photocatalytic TiO2 nanomaterials as potential antimicrobial and antiviral agents: Scope against blocking the SARS-COV-2 spread. Micro Nano Eng..

[128] Augustine R., Rehman S.R., Ahmed R., Zahid A.A., Sharifi M., Falahati M., Hasan A. (2020). Electrospun chitosan membranes containing bioactive and therapeutic agents for enhanced wound healing. Int. J. Biol. Macromol..

[129] Wang W., Xue C., Mao X. (2020). Chitosan: Structural modification, biological activity and application. Int. J. Biol. Macromol..

[130] Ramesh S., Kovelakuntla V., Meyer A.S. (2021). Three-dimensional printing of stimuli-responsive hydrogel with antibacterial activity. Bioprinting.

[131] Wang S., Li R., Qing Y., Wei Y., Wang Q., Zhang T., Sun C., Qin Y., Li D., Yu J. (2019). Antibacterial activity of Ag-incorporated zincosilicate zeolite scaffolds fabricated by additive manufacturing. Inorg. Chem. Commun..

[132] Zhang Y., Zhai D., Xu M., Yao Q., Zhu H., Chang J., Wu C. (2017). 3D-printed bioceramic scaffolds with antibacterial and osteogenic activity. Biofabrication.

[133] Li J., Li L., Zhou J., Zhou Z., Wu X.-L., Wang L., Yao Q. (2019). 3D printed dual-functional biomaterial with self-assembly micro-nano surface and enriched nano argentum for antibacterial and bone regeneration. Appl. Mater. Today.

[134] Wu Z., Hong Y. (2019). Combination of the Silver–Ethylene Interaction and 3D Printing to Develop Antibacterial Superporous Hydrogels for Wound Management. ACS Appl. Mater. Interfaces.

[135] Shi G., Wang Y., Derakhshanfar S., Xu K., Zhong W., Luo G., Liu T., Wang Y., Wu J., Xing M. (2019). Biomimicry of oil infused layer on 3D printed poly (dimethylsiloxane): Non-fouling, antibacterial and promoting infected wound healing. Mater. Sci. Eng. C.

[136] Zhu Y., Liu K., Deng J., Ye J., Ai F., Ouyang H., Wu T., Jia J., Cheng X., Wang X. (2019). 3D printed zirconia ceramic hip joint with precise structure and broad-spectrum antibacterial properties. Int. J. Nanomed..

[137] Zou F., Jiang J., Lv F., Xia X., Ma X. (2020). Preparation of antibacterial and osteoconductive 3D-printed PLGA/Cu(I)@ZIF-8 nanocomposite scaffolds for infected bone repair. J. Nanobiotechnol..

[138] Shao J., Ma J., Lin L., Wang B., Jansen J.A., Walboomers X.F., Zuo Y., Yang F. (2019). Three-Dimensional Printing of Drug-Loaded Scaffolds for Antibacterial and Analgesic Applications. Tissue Eng. Part C Methods.

[139] Lv Y., Li L., Yin P., Lei T. (2020). Synthesis and evaluation of the structural and antibacterial properties of doped copper oxide. Dalton Trans..

[140] Shams S., Ahmad W., Memon A.H., Shams S., Wei Y., Yuan Q., Liang H. (2020). Cu/H 3 BTC MOF as a potential antibacterial therapeutic agent against *Staphylococcus aureus* and *Escherichia coli*. New J. Chem..

[141] Dorj B., Park J.-H., Kim H.-W. (2012). Robocasting chitosan/nanobioactive glass dual-pore structured scaffolds for bone engineering. Mater. Lett..

[142] Zhang D., Liu Y., Liu Z., Wang Q. (2020). Advances in Antibacterial Functionalized Coatings on Mg and Its Alloys for Medical Use—A Review. Coatings.

[143] Dorati R., DeTrizio A., Modena T., Conti B., Benazzo F., Gastaldi G., Genta I. (2017). Biodegradable Scaffolds for Bone Regeneration Combined with Drug-Delivery Systems in Osteomyelitis Therapy. Pharmaceuticals.

[144] Barnes R.J., Molina R., Xu J., Dobson P.J., Thompson I.P. (2013). Comparison of TiO_2_ and ZnO nanoparticles for photocatalytic degradation of methylene blue and the correlated inactivation of gram-positive and gram-negative bacteria. J. Nanopart. Res..

[145] Bandyopadhyay A., Bose S., Das S. (2015). 3D printing of biomaterials. MRS Bull..

[146] Guler S., Ozseker E.E., Akkaya A. (2016). Developing an antibacterial biomaterial. Eur. Polym. J..

[147] Ahmed W., Zhai Z., Gao C. (2019). Adaptive antibacterial biomaterial surfaces and their applications. Mater. Today Bio.

[148] Saberi A., Bakhsheshi-Rad H.R., Ismail A.F., Sharif S., Razzaghi M., Ramakrishna S., Berto F. (2022). The Effect of Co-Encapsulated GO-Cu Nanofillers on Mechanical Properties, Cell Response, and Antibacterial Activities of Mg-Zn Composite. Metals.

[149] Shih Y.-T., Chen A.-P., Lai M.-F., Lin M.-C., Shiu B.-C., Lou C.-W., Lin J.-H. (2022). Hemostasis Evaluation of Antibacterial and Highly Absorbent Composite Wound Dressings in Animal Hemostasis Models. Polymers.

[150] Zhang H., Liu L., Hou P., Pan H., Fu S. (2022). Polyisocyanide Quaternary Ammonium Salts with Exceptionally Star-Shaped Structure for Enhanced Antibacterial Properties. Polymers.

[151] Chaiwarit T., Sommano S.R., Rachtanapun P., Kantrong N., Ruksiriwanich W., Kumpugdee-Vollrath M., Jantrawut P. (2022). Development of Carboxymethyl Chitosan Nanoparticles Prepared by Ultrasound-Assisted Technique for a Clindamycin HCl Carrier. Polymers.

[152] Li Y., Chen Y., Wu Q., Huang J., Zhao Y., Li Q., Wang S. (2022). Improved Hydrophobic, UV Barrier and Antibacterial Properties of Multifunctional PVA Nanocomposite Films Reinforced with Modified Lignin Contained Cellulose Nanofibers. Polymers.

[153] Suvandee W., Teeranachaideekul V., Jeenduang N., Nooeaid P., Makarasen A., Chuenchom L., Techasakul S., Dechtrirat D. (2022). One-Pot and Green Preparation of *Phyllanthus emblica* Extract/Silver Nanoparticles/Polyvinylpyrrolidone Spray-On Dressing. Polymers.

[154] Tang Y., Cao L., Xu L., Wang Z., Shi Q., Zhang Y., Yu L. (2022). Dependable Performance of Thin Film Composite Nanofiltration Membrane Tailored by Capsaicin-Derived Self-Polymer. Polymers.

[155] McFarland A.W., Elumalai A., Miller C.C., Humayun A., Mills D.K. (2022). Effectiveness and Applications of a Metal-Coated HNT/Polylactic Acid Antimicrobial Filtration System. Polymers.

[156] Wang F., Wang R., Pan Y., Du M., Zhao Y., Liu H. (2022). Gelatin/Chitosan Films Incorporated with Curcumin Based on Photodynamic Inactivation Technology for Antibacterial Food Packaging. Polymers.

[157] Elessawy N.A., Gouda M.H., Elnouby M., Ali S.M., Salerno M., Youssef M.E. (2022). Sustainable Microbial and Heavy Metal Reduction in Water Purification Systems Based on PVA/IC Nanofiber Membrane Doped with PANI/GO. Polymers.

[158] Cocean G., Cocean A., Postolachi C., Garofalide S., Bulai G., Munteanu B.S., Cimpoesu N., Cocean I., Gurlui S. (2022). High-Power Laser Deposition of Chitosan Polymers: Medical and Environmental Applications. Polymers.

[159] Tsegay F., Elsherif M., Butt H. (2022). Smart 3D Printed Hydrogel Skin Wound Bandages: A Review. Polymers.

[160] Patti A., Acierno D. (2022). Towards the Sustainability of the Plastic Industry through Biopolymers: Properties and Potential Applications to the Textiles World. Polymers.

[161] Maiz-Fernández S., Pérez-Álvarez L., Silván U., Vilas-Vilela J.L., Lanceros-Méndez S. (2022). pH-Induced 3D Printable Chitosan Hydrogels for Soft Actuation. Polymers.

[162] Jamnongkan T., Jaroensuk O., Khankhuean A., Laobuthee A., Srisawat N., Pangon A., Mongkholrattanasit R., Phuengphai P., Wattanakornsiri A., Huang C.-F. (2022). A Comprehensive Evaluation of Mechanical, Thermal, and Antibacterial Properties of PLA/ZnO Nanoflower Biocomposite Filaments for 3D Printing Application. Polymers.

[163] Gruber P., Hoppe V., Grochowska E., Paleczny J., Junka A., Smolina I., Kurzynowski T. (2021). Material Extrusion-Based Additive Manufacturing of Poly(Lactic Acid) Antibacterial Filaments—A Case Study of Antimicrobial Properties. Polymers.

[164] Idriss H., Elashnikov R., Rimpelová S., Vokatá B., Haušild P., Kolská Z., Lyukatov O., Švorčík V. (2021). Printable Resin Modified by Grafted Silver Nanoparticles for Preparation of Antifouling Microstructures with Antibacterial Effect. Polymers.

[165] Ahmed W., Siraj S., Al-Marzouqi A. (2021). Embracing Additive Manufacturing Technology through Fused Filament Fabrication for Antimicrobial with Enhanced Formulated Materials. Polymers.

[166] Rezić I., Majdak M., Bilić V.L., Pokrovac I., Martinaga L., Škoc M.S., Kosalec I. (2021). Development of Antibacterial Protective Coatings Active against MSSA and MRSA on Biodegradable Polymers. Polymers.

[167] Cho Y.S., Kim H.-K., Ghim M.-S., Hong M.W., Kim Y.Y., Cho Y.-S. (2020). Evaluation of the Antibacterial Activity and Cell Response for 3D-Printed Polycaprolactone/Nanohydroxyapatite Scaffold with Zinc Oxide Coating. Polymers.

[168] Yang F., Zeng J., Long H., Xiao J., Luo Y., Gu J., Zhou W., Wei Y., Dong X. (2020). Micrometer Copper-Zinc Alloy Particles-Reinforced Wood Plastic Composites with High Gloss and Antibacterial Properties for 3D Printing. Polymers.

[169] Hasan K.F., Kóczán Z., Horváth P.G., Bak M., Horváth A., Bejó L., Alpár T. (2022). Green synthesis of nanosilver using Fomes fomentarius mushroom extract over aramid fabrics with improved coloration effects. Text. Res. J..

[170] Hasan K.M.F., Wang H., Mahmud S., Islam A., Habib A., Genyang C. (2022). Enhancing mechanical and antibacterial performances of organic cotton materials with greenly synthesized colored silver nanoparticles. Int. J. Cloth. Sci. Technol..

[171] Hasan K.M.F., Horváth P.G., Alpar T. (2020). Potential Natural Fiber Polymeric Nanobiocomposites: A Review. Polymers.

[172] Sikorski D., Bauer M., Frączyk J., Draczyński Z. (2022). Antibacterial and Antifungal Properties of Modified Chitosan Nonwovens. Polymers.

